# Management, risk factors and treatment outcomes of rhegmatogenous retinal detachment associated with giant retinal tears: scoping review

**DOI:** 10.1186/s40942-024-00552-6

**Published:** 2024-04-23

**Authors:** Miguel A. Quiroz-Reyes, Zaheer-Ud-Din Babar, Rabia Hussain, Zhe Chi Loh, Erick A. Quiroz-Gonzalez, Miguel A. Quiroz-Gonzalez, Virgilio Lima-Gomez

**Affiliations:** 1 Retina Department of Oftalmologia Integral ABC (Nonprofit Medical and Surgical Organization, National Autonomous University of Mexico, Av. Paseo de las Palmas 735 Suite 303, 11000 Lomas de Chapultepec, Mexico City, Mexico; 2 Department of Pharmacy, University of Huddersfield, HD1 3DH Queensgate, Huddersfield, UK; 3 School of Pharmaceutical Sciences, Universiti Sains Malaysia, 11800 Gelugor, Pulau Pinang Malaysia; 4 Institute of Ophthalmology, National Autonomous University of Mexico, Av. Chimalpopoca 14. Col. Obrera, 06800 Mexico City, Mexico; 5 Juarez Hospital, Public Assistance Institution, Av. Politecnico Nacional 5160, Colonia Magdalena de las Salinas, 07760 Mexico City, Mexico

**Keywords:** Giant retinal tear, Scleral buckling, Primary vitrectomy, High myopia, Proliferative vitreoretinopathy, Rhegmatogenous retinal detachment

## Abstract

**Background:**

Rhegmatogenous retinal detachment (RRD) is a serious condition that occurs when the retina detaches from its underlying retinal pigment epithelium. RRDs associated with giant retinal tears (GRTs) are caused by retinal tears at least 90° or one-quarter of the circumferential extent. This scoping review systematically identifies and summarizes clinical studies evaluating surgical techniques for the management of GRT-related RRDs, discusses functional and visual outcomes and the risk factors affecting treatment outcomes.

**Methods:**

This study was conducted in accordance with the Preferred Reporting Items for Systematic Reviews and Meta-Analyses (PRISMA) guidelines. PubMed, Scopus, Google Scholar, and Springer Link databases were searched for relevant papers (from January 2001 to March 2023). Studies that were published in the English language and reported the risk factors, management, and treatment outcomes of GRT-related RRDs were included in the review. The outcome measures included anatomic success rates, changes in BCVA (logMAR) from baseline to the final follow-up, and adverse events.

**Results:**

A total of 11,982 articles were identified. After the title and abstract review, 71 studies were deemed eligible for full-text review. Thirty-six studies that met the eligibility criteria were included in the final review. Four surgical techniques were identified: pars plana vitrectomy (PPV), combined PPV and scleral buckling, scleral buckling alone, and pneumatic retinopexy. Various types of tamponades, including gas, silicone oil, and air, have been used. PPV was the most commonly used surgical technique in 33.1–100% of patients. Among the 20 studies that used PPV alone, 17 were associated with preoperative PVR. In addition, scleral buckling alone or in combination with PPV was reported as a treatment option in 10 studies, with 2–100% of patients experiencing scleral buckling alone and 13.6–100% experiencing combined PPV and complementary scleral buckling. Primary anatomic success (PAS) was achieved with retinal reattachment via a single operation with no residual tamponade, whereas final anatomic success (FAS) was achieved via more than one operation with no residual tamponade. Reported single surgery anatomic success (SSAS) rates range from 65.51 to 100%. The preoperative best-corrected visual acuity (BCVA) ranged from 0.067 to 2.47 logMAR, whereas the postoperative BCVA ranged from 0.08 to 2.3 logMAR. An improvement in visual acuity was observed in 29 studies. Cataracts (3.9-28.3%) were the most common postoperative complication, followed by high IOP (0.01-51.2%) and PVR (0.8-31.57%).

**Conclusion:**

PPV is the most common surgical technique, and currently microincision vitrectomy surgery (MIVS) systems are commonly employed. Silicone oil is the most frequently used tamponade in RRD repair. Risk factors for GRT-related RRD include age, sex, lens status, high myopia status, proliferative vitreoretinopathy (PVR), presenting visual acuity, the extent of the GRT and retinal detachment, and macular involvement. Future research areas include guidelines to reduce variability in the reporting of surgical methodology, choice of tamponades, and reporting of functional and visual outcomes to inform the best therapeutic interventions in GRT-related RRD.

**Supplementary Information:**

The online version contains supplementary material available at 10.1186/s40942-024-00552-6.

## Background

Rhegmatogenous retinal detachment (RRD) is a serious eye condition that occurs when the neurosensory retina detaches from the underlying retinal pigment epithelium (RPE) [1]. This detachment is usually caused by a tear or hole in the retina, resulting in the accumulation of fluid between the retina and the surrounding tissues, causing the fluid to pull away [2]. The prevalence of RRD has increased from 1 in 10,000 to 13 in 10,000 in recent years, and 1.3 times more males than females were found to be affected by this condition [3]. If left untreated, RRD can lead to vision loss or blindness [4]. The risk factors for RRD include age, myopia, previous eye surgery or trauma, and family history [2]. Although RRD can be treated with surgery, the treatment outcome depends on disease severity and the speed of diagnosis and management [5].

Giant retinal tear (GRT)-related RRDs are a type of retinal detachment characterized by full-thickness tears in the retina at least 90° or one-quarter of the circumferential extent. GRTs account for approximately 1.5% of RRD cases, and they are more common in males comprising 72% of all GRT cases [6]. GRTs usually occur spontaneously, but certain risk factors such as trauma, young age, high myopia, and hereditary conditions such as Marfan syndrome and Stickler syndrome may also be involved [6–8]. GRTs are considered high-risk factors for RRD. If not treated promptly, it may lead to extensive and complex retinal detachment, proliferative vitreoretinopathy (PVR), and poor visual outcomes [9, 10].

The management of GRT-related RRDs depends on the size, location, extent, and severity of both the GRT and RRD and includes several techniques, such as scleral buckling, pneumatic retinopexy, fluid-air exchange, combined scleral buckling and vitrectomy, and primary pars plana vitrectomy (PPV) involving gas or silicone oil tamponade. However, owing to the high risks of intraoperative and postoperative complications and technical difficulties, GRT-related RRDs pose a great challenge to treatment outcomes [6].

Previous studies have classified RRDs based on functional outcomes or have focused on the differences in the clinical outcomes of GRT-related RRDs [11]. Despite high primary anatomical success (PAS) and final anatomical success (FAS) rates, the final visual outcome may be limited owing to the postoperative complications of retinal detachment repair, including the formation of postoperative PVR and epiretinal membrane [11, 12]. GRT-related RRDs are surgically challenging to manage because of the frequent rolling of the posterior edge of the retinal flap and the high incidence of PVR, which increases the risk of redetachment [13, 14]. The advent of low-viscosity perfluorocarbon liquids (PFCLs) with specific gravities greater than that of water has facilitated surgical reapplication of the retina assisting in the displacement of the subretinal fluid (SRF) and revolutionizing the management of complicated retinal detachments [13, 14]. Additional innovations consistent with small-gauge microincision vitrectomy surgery (MIVS), faster-speed cutters, wide-angle viewing systems (WAVS), and laser retinopexy in combination with the use of silicone oil have further improved treatment outcomes, reaching a single surgical anatomic success (SSAS) rate between 81.8% and 100% [14, 15].

To the best of our knowledge, no scoping review has been conducted on the risk factors, management strategies, or treatment outcomes of patients with GRT-related RRDs. Therefore, this systematic review aimed to summarize the surgical techniques involved in the management of GRT-related RRDs, functional and visual outcomes, and the risk factors affecting treatment outcomes. Furthermore, we performed a comprehensive analysis of the postoperative complications. The results of this study provide valuable resources for understanding the most suitable surgical techniques for adults with GRT-related RRDs. Additionally, this review highlights areas that require further investigation, potentially encouraging future research and innovation to manage these visually debilitating conditions.

## Methods

### Information sources and search strategy

This systematic review was conducted in accordance with the Preferred Reporting Items for Systematic Reviews and Meta-Analyses (PRISMA) guidelines [16, 17]. The search strategy included a combination of keywords and Medical Subject Headings (MeSH) terms related to GRTs, surgical approaches, functional outcomes, and complications. The study protocol was registered with PROSPERO under registration number CRD42023401049, which can be found at https://www.crd.york.ac.uk/prospero/display_record.php?RecordID=401049. The literature was searched by utilizing Boolean operators such as “AND” or “OR” to combine the following keywords and MeSH terms: *giant retinal tears OR GRT AND rhegmatogenous retinal detachment OR RRD AND surgical approaches OR complications OR pars plana vitrectomy OR PPV OR scleral buckling OR scleral buckling OR tamponade OR gas OR silicone OR best visual acuity OR BVA.* The combinations were searched using the PubMed platform, and this step was repeated using databases such as Scopus, Google Scholar, and Springer Link from January 2001 to March 2023. These databases were selected because of their easy accessibility and availability of publications on the topic [18]. The reference lists of relevant studies were also reviewed to avoid missing information and to identify related articles.

### Selection process

Articles were searched based on the Population, Intervention, Comparison, and Outcome (PICO) framework. The PICO framework for this study is presented in Table 1, and the search strategies applied to the different databases are listed in Table S1 (Appendix 1 in the Supplementary file). The inclusion criteria were original research articles of any study design related to GRT-related RRDs published in full text and in English language between January 2001 and March 2023. Randomized controlled trials (RCTs), prospective cohort studies, retrospective cohort studies, case reports, and case series were considered eligible for inclusion. Commentaries, review articles, editorials, letters to the editor, and conference proceedings were excluded. Studies that reported RRD caused by retinal tears other than GRT and pediatric case reports were also excluded.

**Table 1:** PICO framework for the study

Population	Studies which included patients ≥ 18 years old and surgically managed GRT-related RRD and reported at least one of the following outcomes: anatomical outcomes or functional outcomes.
Intervention	Surgical procedures to treat GRT-related RRD. scleral buckle; PPV; Combined PPV/scleral buckling; PPV with tamponade gas; PPV with silicone oil; Photocoagulation and scleral buckling; Primary PPV without scleral buckling, and pneumatic retinopexy.
Comparator	Not applicable. No specific comparison is needed.
Outcome	Anatomic success: SSAS rate, PAS rate, overall success rate, and FAS rate, which will be all reported as percentages. Functional success: Change in BCVA from baseline (logMAR units) to final follow-up. List of complications and risk factors: Number, type and severity of postoperative complications, common adverse events, and types of tamponades.

BCVA, best-corrected visual acuity; FAS, final anatomic success; GRT, giant retinal tear; logMAR, logarithm of the minimum angle of resolution; PPV, pars plana vitrectomy; PICO, population, intervention, comparator, outcomes; PAS, primary anatomic success; RRD, rhegmatogenous retinal detachment; SSAS, single surgical anatomic success

**Fig. 1:**
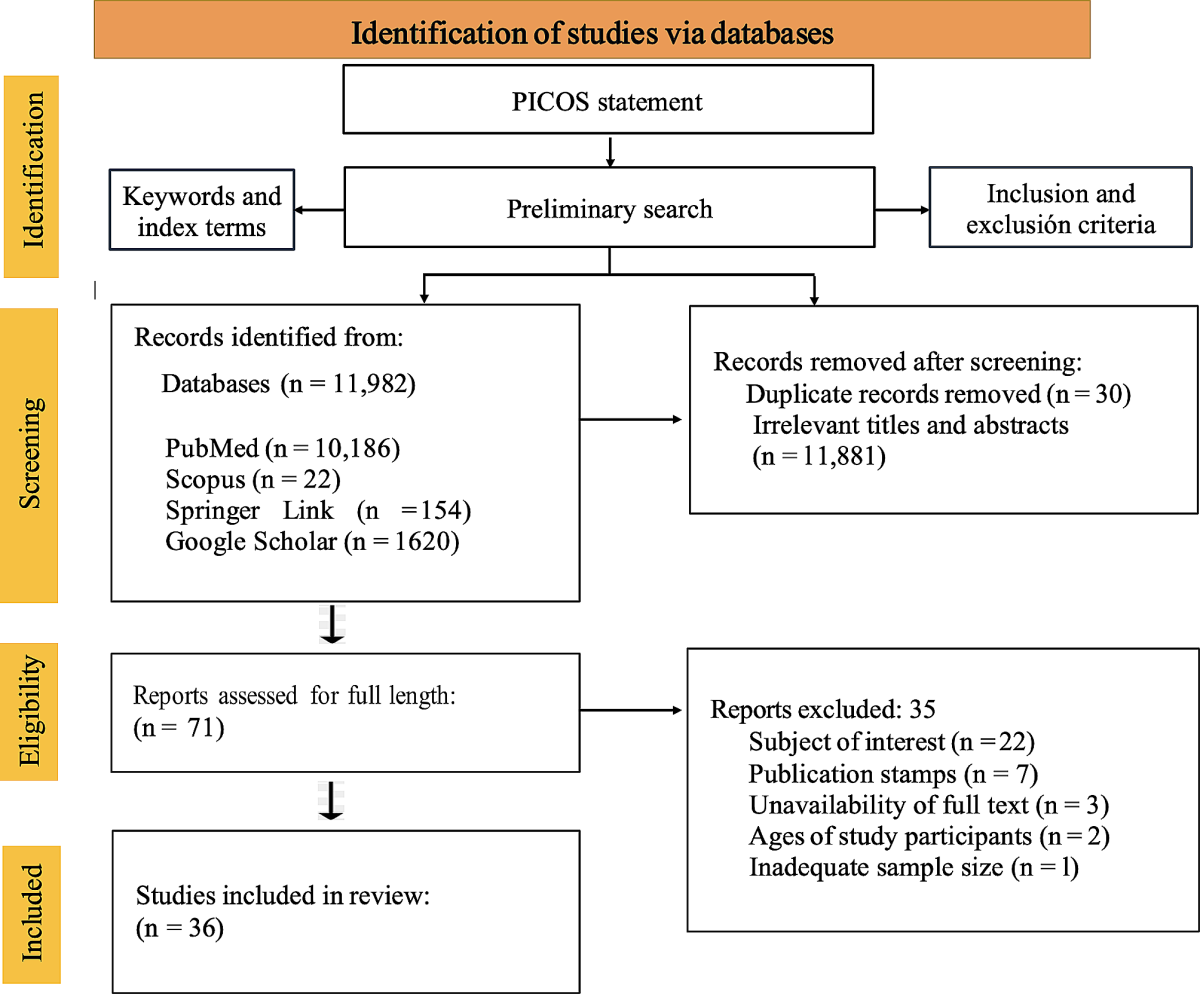
PRISMA flow diagram showing the search strategy with detailed screening and selection of studies

Two authors (RH and ZCL) independently screened the studies for eligibility, and a third author (ZUB) resolved any conflicts. The titles and abstracts of original peer-reviewed research studies published in English were identified from various databases [19–21]. Full-text articles were assessed when the titles and abstracts were insufficient to provide relevant information. Full-text articles that did not meet the inclusion criteria were also excluded. The studies were selected based on their relevance and acceptability [22, 23]. Furthermore, an explicit method that emphasizes predefined inclusion and exclusion criteria was strictly applied to ensure the quality of the selection process [24]. Commentaries, review articles, letters to editors, and conference proceedings were also excluded. The identification of the studies via databases and the PRISMA flow diagram are shown in Fig. 1.

### Data collection

The retrieved articles were uploaded to the Covidence database for screening. Data were extracted by two independent authors (RH and ZCL) on a data extraction sheet using Microsoft Excel®. The titles and abstracts of the identified articles were screened for eligibility based on the inclusion criteria. The information extracted from the selected studies, including country, study design, setting, inclusion criteria, exclusion criteria, surgical repair, comparison, presence of GRT, postoperative follow-up period, number of patients, number of eyes, functional and anatomical outcomes, associated risk factors, and recommendations, was evaluated for final inclusion by two independent authors (MAQR and VLG).

### Quality assessment

Two authors (ZCL and RH) independently assessed the quality of the included studies. Case series and cohort studies were evaluated using the Joanna Briggs Institute (JBI) checklist, which comprises 10 and 11 criteria, respectively [25, 26]. One point was allocated for each criterion, and zero points were allocated for criteria that were not satisfied [27–30]. A higher score indicates a better-quality study [30]. Disagreements were resolved by discussion and consensus.

### Data synthesis and analysis

All studies included in this research were evaluated through thematic analysis to synthesize key findings from the data [31–33]. Thematic analysis involves reading texts and identifying key findings that capture the overall meaning of the text [34, 35]. The key characteristics of the included studies were synthesized in the form of information such as country, study design, setting, inclusion criteria, exclusion criteria, surgical repair of retinal detachment, comparison, presence of GRT, postoperative follow-up period, number of patients, and number of eyes. The statistical data were included to indicate the magnitude of the key findings but were not used in the data synthesis process. Therefore, missing summary statistics were not required in this systematic review.

## Results

After screening 11,982 titles and abstracts from PubMed, Scopus, Springer Link, and Google Scholar, 71 studies underwent full-length assessment after duplication removal using the Endnote software. Fifteen studies reported data in Asia (South Korea, Singapore, Iran, China, Israel, Bangladesh, Turkey, Thailand, and Saudi Arabia), 12 in Europe (Slovenia, the United Kingdom, Germany, Ireland, Italy, Norway, the Netherlands, and Denmark), five in North America (the United States and Mexico), one in South America, one in Africa (Nigeria), and one in Oceania (Australia) [10, 36–69]. One study included data from 48 countries across 5 continents [70].

A total of 36 studies were included in the review [10, 36–70]. The reasons for exclusion included subjects of interest (*n* = 22), publication status (*n* = 7), unavailability of the full text (*n* = 3), participant age (*n* = 2), and inadequate sample size (*n* = 1) (Fig. 1). The detailed characteristics of the studies are listed in Table S2 (Appendix 2 in the Supplementary file). Additional relevant information such as the mean age, preoperative and postoperative PVR, SSAS rate, PAS rate, FAS rate, important definitions, occurrence, type of complications, identified risk factors, and conclusion are listed in Table S3 (Appendix 3 in the Supplementary file). Of the 36 studies that fulfilled the eligibility criteria, 21 were case series studies and 15 were cohort studies. According to Table S4 (Appendix 4 in the Supplementary file), the quality assessment of the 21-case series revealed that 20 studies scored nine points, whereas the remaining study scored eight points. Likewise, the quality assessment of the 15 cohort studies revealed one study with a score of 11, nine studies with a score of 10, four studies with a score of nine, and one study with a score of eight, as indicated in Table S5 (Appendix 5 in the Supplementary file).

### Surgical techniques, tamponades, anatomic and functional results, risk factors and postoperative complications

Four surgical techniques were identified: PPV alone, combined PPV and scleral buckling, scleral buckling alone, and pneumatic retinopexy. PPV was the most commonly used surgical technique, ranging between 33.1% and 100% in 34 studies [10, 36–56, 58, 59, 61–70] (Appendix 2 in the Supplementary file). Additionally, 13 studies included only patients with RRD treated with PPV [45, 46, 49, 50, 53, 55, 56, 59, 61, 64, 67–69]. Among the 20 studies that only used PPV to manage GRT-related RRD, 17 were associated with preoperative PVR [41, 42, 44–47, 49, 50, 53, 55, 56, 58, 59, 61, 62, 64, 66–69]. In addition, scleral buckling alone or in combination with PPV was reported as a treatment option in 10 studies, with prevalence rates ranging from 2 to 100% for scleral buckling alone and from 13.6 to 100% for combined PPV and complementary scleral buckling [10, 36–39, 43, 48, 51, 52, 54, 57, 60, 63, 70]. One study exclusively employed scleral buckling as the surgical technique in all patients [60]. Another study employed a combination of scleral buckling and PPV as a surgical technique in all patients [51]. Only three studies mentioned the use of pneumatic retinopexy for surgical management, with 0.4%, 5.0%, and 100% (all primary surgeries) usage rates [39, 44, 52].

Retinal detachment repair commonly involves the use of tamponades, including gas, silicone oil, and air tamponade. Among these options, silicone oil is the most frequently used tamponade, as reported in 22 studies, followed by gas and air tamponades in 15 and 6 studies, respectively [10, 36, 38, 40–43, 48, 50, 52–59, 61, 62, 64–67]. The use of gas tamponade ranges from 0.9 to 100% of the cases, whereas the use of silicone oil tamponade varies from 3.5 to 100% [10, 36, 37, 40–43, 48, 50, 52–55, 57–59, 61, 62, 64–67]. Similarly, the use of air tamponade ranges from 0.3 to 100% [50, 52, 54, 56, 58, 66]. In addition, 12 studies have reported the use of PFCL to flatten and stabilize the retina [10, 38, 42, 45, 47, 51, 55, 58, 59, 61, 64, 68].

Anatomic success refers to the complete reattachment of the retina achieved through one or more surgeries [10, 36–39, 41, 43, 44, 46, 48, 49, 52, 59, 63, 64, 67–69]. PAS was achieved with retinal reattachment via a single operation and no residual tamponade, whereas FAS was achieved via more than one operation and no residual tamponade [10, 36–39, 41, 43, 44, 46, 48, 49, 52, 59, 63, 64, 67–69]. The reported SSAS rates ranged from 65.51 to 100%, with the majority falling in the 80–90% range, whereas the FAS rate reached 100% [10, 36–41, 43–46, 48–52, 54–60, 63–68].. The highest primary SSAS rate (100%) was reported in a study from India, whereas four studies from South Korea, the United States of America, Israel, and Columbia reported FAS rates of 100% [10, 36, 55, 57, 59]. Twenty studies reported an improvement in the FAS score compared with the PAS score [10, 36–41, 43, 46, 48, 50, 52, 54, 56–58, 60, 64, 65, 67].

The success of the visual outcome was determined by the change in visual acuity from baseline to the most recent follow-up [10, 37–39, 43, 48, 59, 63, 69]. The preoperative best-corrected visual acuity (BCVA) ranged from 0.067 logMAR (logarithm of the minimum angle of resolution) to 2.47 logMAR, whereas the postoperative BCVA ranged from 0.08 logMAR to 2.3 logMAR [10, 36–40, 42, 43, 45–51, 53, 54, 57–58, 61–69]. An improvement in visual acuity was observed in 29 studies that compared the preoperative and postoperative visual acuity data [10, 36–40, 42, 43, 45–51, 53, 54, 56–58, 61–69] (Table S3, Appendix 3).

Several risk factors for poor functional outcomes and complications in patients with GRT-related RRDs are listed in Table S3 (Appendix 3 in the Supplementary file). The risk factors identified included age, sex, lens status, high myopia status, PVR, presenting visual acuity, worse visual acuity, preoperative detached macula, a greater extent of GRT (150º), and extent of detachment [10, 36, 38–41, 43, 46, 48, 49, 52, 54, 57, 63, 67, 70]. However, PVR was the most commonly identified risk factor, followed by age and the number of detached retinal quadrants, as discussed in 10, six, and four studies, respectively [10, 36, 38–41, 43, 46, 48, 49, 52, 54, 57, 63, 67, 70].

Postoperative complications included new retinal breaks, PVR, macular holes, cataracts, epiretinal membrane formation, macular pucker, increased intraocular pressure (IOP), subretinal hemorrhage, iatrogenic tears, suprachoroidal hemorrhage, aborted buckling, irritation requiring intervention, hypotony, PFCL emulsification, endophthalmitis, eye movement disorder, macular edema, PFCL in the anterior chamber, serous choroidal detachment, lens trauma, retinal detachment with inferior traction, persistent corneal epithelial defects, corneal decompensation, secondary glaucoma, choroidal detachment, orbital cellulitis, silicone oil in the anterior chamber, retinal vessel printing or retinal displacement, granulomatous inflammation, and optic nerve atrophy [10, 36, 38–40, 43, 45, 47, 54–56, 58–61, 64, 65, 67–69]. Among these, cataracts (3.9-28.3%) were the most commonly reported, followed by IOP (0.01-51.2%), PVR (0.8-31.57%), and epiretinal membrane or macula pucker formation (1.7-33.3%), as reported in 11, 10, eight, and seven studies, respectively [10, 36, 38–40, 45, 47, 54–56, 58, 59, 61, 64, 65, 67–69].

## Discussion

Given the complexity of the various factors involved in disease causation and treatment outcomes, the present study aimed to summarize the risk factors, management options and treatment outcomes of RRD associated with GRT. A thorough search identified a vast body of literature related to this complex condition. Interestingly, the literature is dominated by retrospective studies and case series. Our primary goal was to delve into more comprehensive and rigorous evidence, particularly seeking randomized and comparative studies that could offer insights into the comparative effectiveness of various surgical interventions. However, the limited availability of such studies prompted us to broaden our search criteria to encompass all relevant studies conducted since 2001. This strategic expansion allowed us to compile a comprehensive overview of the literature on GRT management, although it predominantly comprised of retrospective and case series studies. This approach allowed us to integrate and analyze the available data in detail, shedding light on the state of research on GRT-related RRD management over the past two decades and potential directions for future research on the surgical management of GRT-related RRDs.

This review investigated the surgical techniques used to treat GRT-related RRDs. The literature shows that GRT-related RRDs are mostly treated with PPVusing MIVS. These findings are consistent with those of a study by Li et al. (2021) in which 83% of GRT-related RRDs were treated with PPV alone [10]. Another study analyzed data from 751 eyes and revealed that the PPV accounted for 89% (*n* = 668) of eyes, resulting in a 91.2% SSAS rate in initial surgeries and a functional success rate of 96.7% in patients using MIVs [71].

The use of scleral buckling alone or in combination with PPV as a treatment option has been reported in a small number of studies, and its prevalence varies widely [75]. A retrospective study by Rodriguez et al. (2022) highlighted that 76% of combined scleral buckling and PPV procedures resulted in favorable anatomical and functional outcomes [76]. These findings contrast with the results reported by Moinuddin et al. (2021), as only 6.8% of patients were treated with combined scleral buckling and PPV, resulting in an 84.3% SSAS rate and a 94.1% FAS rate [71]. This finding suggests that the decision to use scleral buckling as a surgical technique may depend on the surgeon’s preference, the severity of detachment, and the presence or absence of associated risk factors [75].

Our review included only a small number of studies using pneumatic retinopexy for surgical management. Despite being a cost-effective and simple technique compared with PPV and scleral buckling, the infrequent use of pneumatic retinopexy as a surgical management option for GRT-associated RRDs raises questions regarding its viability in this patient population [77]. However, a high PAS rate of 90% can be achieved if appropriate patient selection is performed [78]. Although pneumatic retinopexy therapy has been shown to be effective for certain subtypes of RRD, it may have limited usefulness in patients with GRT-associated RRD because of the potential for repeated gas injections and the risk of complications [79].

Retinal detachment is a serious ocular condition that, if left untreated, may result in permanent vision loss, thus requiring prompt treatment [80]. Tamponades such as air, silicone oil, gas, and PFCLs are vital components of the procedure for successful repair of retinal detachments [81]. The selection of tamponade depends on the surgeon’s preference and patient’s condition. As indicated in our study, silicone oil is the most commonly used tamponade in surgery for treating GRT-related RRD [82]. This is probably because it has a persistent tamponade action and lowers the possibility of recurrent retinal detachment [81]. However, there are several risks associated with the use of silicone oil tamponade, including the development of cataracts, glaucoma, and oil emulsification [83].

Gas tamponade is another popular option; depending on the patient’s condition and surgeon’s preference, a particular gas may be employed. For instance, sulfur hexafluoride (SF_6_) gas is frequently utilized for initial repair, whereas perfluoropropane gas (C_3_F_8_) is favored for repeated applications [1, 81]. Air tamponade is less frequently used than gas or silicone oil; however, owing to its accessibility and lack of the same risks as silicone oil, air tamponade is a desirable option for some patients [1, 84]. Moreover, the retina was flattened and stabilized using the PFCL during surgery. PFCL can also act as a tamponade for a short period; however, this approach is not a permanent repair method. The PFCL is typically removed at the end of surgery, and another tamponade is used to prevent recurrent retinal detachment [85, 86].

In retinal detachment surgery, anatomic success is a crucial indicator of success since it has a direct impact on patient function and visual recovery. The reported rates of anatomic success vary significantly between studies [87]. Moreover, in the majority of studies included in the present review, secondary FAS rates were greater than primary SSAS rates [41–88]. One study suggested that tailoring treatment to the specific needs of each patient can help surgeons optimize anatomic success rates and improve anatomical outcomes in patients with retinal detachment [89].

Functional visual outcome is a major indicator for surgical procedures, as it directly affects the patient’s quality of life [90]. In the included studies, visual acuity improved from baseline to the most recent follow-up visit. Rodriguez et al. (2023) reported that better BCVA was observed in patients who underwent retinal detachment repair within two days of macular detachment than in those with more than three days of macular detachment [91]. Similarly, another study from Switzerland measured BCVA in 56 patients with an attached retina, and 16 (29%) had BCVA > 20/40. However, the presence of PVR and the use of scleral buckling or silicone oil significantly affect the final BCVA [92]. The preoperative visual acuity ranged substantially between studies, from 0.067 to 2.47 logMAR units, indicating that patients with a wide range of visual impairments were included in the analysis. Additionally, several variables, including the underlying causes of retinal detachment and the severity and duration of detachment before surgery, could affect the functional visual outcome [93].

Additionally, a paradigm change occurred in the management of GRT-related RRD after the introduction of the PFCL, which has proven to be a crucial tool for flattening and stabilizing the retina [13–15, 94]. This transformation is marked by a significant shift in surgical techniques and outcomes [6, 13–15]. In the pre-PFCL era, retinal detachment repair for GRTs relied primarily on conventional methods such as PPV alone or scleral buckling. With the adoption of PFCLs, there was a notable improvement in the primary SSAS rate, which consistently ranged from 80 to 90% [6, 7]. Furthermore, the FAS rate is consistently between 94% and 100% [6, 7].

Based on these findings, PVR was the most frequently identified risk factor influencing anatomical and functional success, followed by more than 150º of GRT extent, number of detached retinal quadrants, preoperative visual acuity, preoperative macular status, high myopia status and age [10, 36, 38–41, 43, 46, 48, 49, 52, 54, 57, 63, 67, 70]. PVR is caused by cellular membrane growth and contraction within the vitreous cavity and on both sides of the retinal surface and can cause intraretinal fibrosis [95]. It is a common cause of retinal detachment repair failure affecting 0.8–31.57% of patients [10, 36, 38, 40, 47, 54, 58, 59, 96, 97].

Smaller-gauge transconjunctival microincision vitrectomy surgery (MIVS) may reduce postoperative inflammation and scarring but does not lead to superior outcomes [95, 98, 99]. Age is also an important consideration, as a study showed that patients aged ≥ 35 years had a greater risk of primary anatomical failure following scleral buckling surgery for uncomplicated RRD [100]. Another study of patients aged ≥ 85 years who underwent vitrectomy revealed that more than half (52%) of them developed multiple complications, including choroidal detachment, subretinal hemorrhage, and macular holes [101]. However, a contrasting study claimed that age did not have a significant impact on the rate of repeated PPV [40]. In addition, the number of detached retinal quadrants affects the success of RRD repair, with more quadrants leading to more extensive detachment and challenging repair [102, 103].

This scoping review provides crucial insights into the postoperative complications associated with GRT-related RRD after retinal reattachment surgery. Cataracts were the most commonly reported complications with an incidence ranging from 3.9 to 28.3% [38, 39, 54–56, 58, 59, 64, 65, 68, 69]. A meta-analysis of RCTs demonstrated that postoperative cataract progression occurred in 55.1% of 477 eyes treated for RRD [12]. PPV was more frequently associated with postoperative cataracts (53.1%) than scleral buckling management (23.6%); however, another study reported that the rate of cataract progression was 4.11 times greater in the PPV group than in the scleral buckling group [12, 102]. Cataracts commonly develop after retinal reattachment surgery because of the manipulation of eye structures. The contributing factors include retrolental vitreous removal, nuclear sclerosis, light toxicity, oxidation of lens proteins, silicone oil or intravitreal gas use, intraoperative mechanical trauma, and prolonged exposure to irrigating solution [12, 104, 105]. However, the use of the smaller-gauge transconjunctival sutureless MIVS technique reduced the risk of cataract development [104, 106].

Studies from Singapore, the United Kingdom, Ireland, Japan, Israel, Italy, Turkey, the Netherlands, Australia, and Thailand have shown that IOP increases by 0.01–51.2% [38, 39, 43, 54, 55, 61, 64, 67–69]. For scleral buckling procedures, the intraoperative IOP increases by 1.4–4.4%, whereas for PPV with silicone oil injection, it increases by 4.8–48% [107]. Choroidal effusion, which causes swelling and anterior rotation of the ciliary body as well as forward shifting of the lens–iris diaphragm, is a common cause of increased IOP [107]. Therefore, patients are advised to maintain a face-down posture to prevent forward displacement of the iris-lens diaphragm [107–109]. A study reported that in situations where there is a high likelihood of postoperative PVR, the combination of vitrectomy and scleral buckling yielded superior anatomical outcomes compared with vitrectomy alone [110].

PVR is a frequent complication of retinal detachment surgery and a major cause of redetachment (Ang et al. (2009) (5) and Ong et al. (2022)) (25). Most PVR cases (approximately 77%) occurred within one month after surgery, whereas 95% of PVR cases occurred within 45 days [94, 96, 111]. Various factors can contribute to the development of PVR, including the presence of new or unsealed retinal breaks that release inflammatory and growth factors that promote cell proliferation in the vitreous cavity as well as surgical manipulation of the retina and vitreous that can stimulate cell migration [94]. Additionally, preexisting ocular conditions such as uveitis or ocular trauma may increase the risk of PVR [94]. Intraoperative adjuvants such as antiproliferative agents (e.g., 5-fluorouracil, daunorubicin, and colchicine) are sometimes used to prevent the formation of ectopic cell sheets and epiretinal membranes [112].

Postoperative epiretinal membrane formation, also known as macular pucker, is another reported complication affecting between 1.7% and 33.3% of patients [38, 45, 54, 56, 64, 67, 69]. In a study that examined the incidence of epiretinal membrane formation after successful primary RRD repair, postoperative epiretinal membranes developed in 15% and 28.5% of patients in the scleral buckling and PPV groups, respectively [113]. Similarly, a meta-analysis revealed that the incidence of epiretinal membrane formation was 7.1% and 5.2% in patients treated with PPV and scleral buckling alone, respectively [114, 115]. Evidence suggests that peeling the ILM during surgery can reduce the likelihood of epiretinal membrane development [115].

PPV with temporary PFCL tamponade is effective in repairing GRT, but it may lead to several complications. The primary complications identified in this review were cataracts and a foreign body response. Notably, postoperative cataract formation has emerged as a predominant concern, and its association with PFCL tamponade has been consistently observed in various studies. This observation aligns with prior research [116], which established a significant link between PFCL use and diverse postoperative complications including hypotony, macular detachment, and cataract formation. Moreover, two distinct studies [117, 118] reported occurrences of foreign body response specifically linked to the use of perfluoro-n-octane (PFO) as postoperative tamponade. Of particular concern was the study by Randolph et al. [118], who reported a 30.4% incidence of foreign body response, which was notably greater than that reported in previous clinical studies that implemented postoperative PFO for retinal detachment [119, 120]. These findings highlight the importance of careful consideration and evaluation of the choice of tamponade agent in retinal surgeries, particularly when PFCLs are used. The identified complications, particularly postoperative hypotony, cataract formation and foreign body response, emphasize the need for ongoing vigilance in refining surgical techniques and optimizing patient outcomes. These insights contribute to a broader understanding of the intricacies associated with the use of tamponade in retinal surgeries and provide a basis for future research aimed at enhancing the safety and efficacy of these procedures.

In summary, it remains uncertain whether there is any potential advantage in the treatment of GRTs by combining PPV with scleral buckling or by using PPV alongside pneumatic retinopexy alone. The analysis of postoperative complications revealed various outcomes. According to Al-Khairi et al., scleral buckling decreases the risk of recurrent retinal detachment [121]. Ramamurthy et al. [122] emphasized the factors leading to poor anatomical success in patients aged < 16 years with visual acuity ≤ 20/400.

Consistent with our findings, Gutierrez et al. [123] and Shunmugam et al. [15] reported that it is difficult to determine whether using scleral buckling combined with PPV for GRTs can affect the outcomes of GRT-associated RRD surgery because of incomplete reporting in two RCTs published in abstract formats. Because evidence from RCTs is lacking, it is difficult to reach conclusions from nonrandomized studies, for which patient characteristics and comparisons of surgical techniques cannot be established.

GRT-related RRDs are rare vitreoretinal conditions that make this surgical approach challenging for retinal surgeons [123]. Most available research data originate from nonrandomized studies, with reported primary retinal reattachment rates ranging from 71.7 to 100% [7, 59, 67, 124–129]. However, 100% final reattachment was achieved by Bhardwaj et al. [128] in a very small number of patients with GRT treated with transscleral diode laser retinopexy using PFO. In contrast, Ramamurthy et al. [122] reported anatomical success in 64% (255 eyes) of the eyes after primary surgery, which improved to 78% (308 eyes) after undergoing a second vitreoretinal procedure for recurrent retinal detachment (53 eyes). These findings indicate that anatomical success varies widely among studies, highlighting the need for further investigation into the factors influencing the outcomes of GRT surgeries. Several factors may contribute to the observed variability in the anatomical success rates. Patient-specific characteristics such as age, as reported by Mehdizadeh et al. [130], and the extent of GRT may play a critical role in determining the success of surgical interventions [131]. Additionally, surgical technique variability, postoperative tamponade, and the use of adjunctive therapies may contribute to the reported differences in outcomes [131].

Despite several interesting findings, this study had several limitations. The primary limitation was the absence of strong evidence from RCTs, which limited the information obtained from the present study. Second, retrospective and nonrandomized prospective studies were predominant in the literature. However, such studies provide useful information about this trend, which is useful, and therefore were included in the present review. Finally, there was a lack of studies that directly compared the different surgical approaches, which would have provided greater evidence. Notwithstanding these limitations, this review revealed that PPV is considered the gold standard approach for treating GRT in adults and achieves better anatomical and functional visual outcomes as long as there is careful postoperative care to overcome postoperative complications.

To enhance our understanding of the impact of combining scleral buckling with vitrectomy, it is essential to consider patient-specific characteristics, GRT attributes (extension, location, and etiology), surgical duration, and the presence of PVR. These studies should incorporate randomization and stratification based on various GRT characteristics, including extension (90°, 90°–180°, and > 180°), location (oral, anterior, or posterior to the equator), PVR stage, and choice of the endotamponade. These analyses should include short-term (three–six months) and long-term (one–two years) outcomes, evaluation of primary retinal reattachment rates, mean changes in BCVA, need for secondary surgeries for retinal reattachment in the eyes of participants, occurrence of adverse events, such as intraocular pressure elevation > 21 mmHg, choroidal detachment, cystoid macular edema, macular pucker, PVR progression, cataract progression in initially phakic eyes, and any other reported adverse events.

### Conclusion and future directions

This review systematically explored the breadth of the literature on the surgical management of GRT-related RRDs in adults. Among published studies, there is considerable variability in the reporting of participant characteristics and eligibility criteria, surgical technique (e.g., PPV alone, combined PPV and scleral buckling, scleral buckling alone, and pneumatic retinopexy), choice of tamponade (e.g., gas, silicone oil, and air tamponade), and definitions of functional and anatomical successes, making it challenging to assimilate data and draw valid comparisons between studies. The development of uniform reporting guidelines following consensus reports (e.g., international task force) could reduce biases in study designs and outcome reporting, which would help to better compare studies on this topic.

Research to date has shown that PPV using MIVS is the most favorable surgical approach. The most frequently observed postoperative complication was cataract formation. However, the use of MIVS reduces the risk of cataract development. PVR and epiretinal membrane formation are frequent complications and major causes of redetachment. Preoperative PVR is the most frequently identified risk factor influencing anatomical and functional success, followed by more than 150° of GRT extent, number of detached retinal quadrants, preoperative visual acuity, preoperative macular status, and high myopia status. The male predominance and the effect of age on surgical success highlight the need for future studies to consider patient-specific management strategies.

Future studies, including studies on age and disease severity factors, are needed to better characterize individual cases and determine whether the results are generalizable to the population of patients. More RCTs are needed to guide clinicians in selecting better therapeutic options for managing GRT-related RRDs.

### Electronic supplementary material

Below is the link to the electronic supplementary material.

Supplementary Material 1

Supplementary Material 2

## Data Availability

The datasets used in this study have been included in the main text. Photographs and figures from this study may be released via a written application to the Photographic Laboratory and Clinical Archives Retina Department of Oftalmologia Integral ABC (nonprofit Medical and Surgical Organization), Av. Paseo de las Palmas 735 suite 303, Lomas de Chapultepec, Mexico City 11000, Mexico and the corresponding author upon request.

## Declarations

### Supplementary Information

Photographs and tables from this study may be released via a written application to the Photographic Laboratory and Clinical Archives of the Retina Department of Oftalmologia Integral ABC, Nonprofit Medical and Surgical Assistance Institute Av. Paseo de las Palmas 735 suite 303, Lomas de Chapultepec, Mexico City 11,000, Mexico, and the corresponding author upon request. All the analysis files can be found in the following supplementary information file (appendixes 1 to 5): For additional information regarding Prospero, please enter to the link https://www.crd.york.ac.uk/prospero/display_record.php?RecordID=401049.

This study adhered to the tenets of the Declaration of Helsinki and received full approval from the appropriate research ethics committee, institutional review committee, and institutional teaching department (the institution did not provide reference numbers for meta-analyses).

## Abbreviations

AL: Axial length
BCVA: Best-corrected visual acuity
CF: Counting fingers
C_2_F_6_: Hexafluoroethane
C_10_F_18_: Perfluorodecalin
C_3_F_8_: Perfluoropropane
DF: Double filling
ERM: Epiretinal membrane
ETDRS: Early treatment diabetic retinopathy study
FAS: Final anatomical success
g: Gauge
GRT: Giant retinal tear
HM: Hand movements
ILM: Internal limiting membrane
IOP: Intraocular pressure
JBI: Joanna Briggs Institute
logMAR: Logarithm of the minimum angle of resolution
LP: Light perception
MIVS: Microincision vitrectomy surgery
MH: Macular hole
MHRD: Macular hole retinal detachment
MeSH: Medical Subject Headings
PARR: Primary anatomical reattachment rate
PAS: Primary anatomical success
PCIOL: Posterior chamber intraocular lens
PDR: Proliferative diabetic retinopathy
PFCL: Perfluorocarbon liquid
PFD: Perfluorodecalin
PPV: Pars plana vitrectomy
PICO: Population, intervention, comparator, outcomes
PFO: Perfluoro-n-octane
PVR: Proliferative vitreoretinopathy
PDMS: Polidimetilsiloxane
PRISMA: Preferred Reporting Items for Systematic Reviews and Meta-Analyses
RD: Retinal detachment
RRD: Rhegmatogenous retinal detachment
SB: Scleral buckling
SD: Standard deviation
SF_6_: Sulfur hexafluoride
SO: Silicone oil
SRF: Subretinal fluid
SSAS: Single surgical anatomic success
VA: Visual acuity
VWTPL: Vitrectomy without the intraoperative use of perfluorocarbon liquid
WAVS: Wide-angle viewing system
