# Supplementary material for: Management, risk factors and treatment outcomes of rhegmatogenous retinal detachment associated with giant retinal tears: scoping review
Source: Int J Retina Vitreous. 2024 Apr 23;10:35. doi: 10.1186/s40942-024-00552-6 (PMC11036595; doi:10.1186/s40942-024-00552-6)
Supplement: Supplementary file 1 — Supplementary Material 1 [file 40942_2024_552_MOESM1_ESM.pdf]

NHS
National Institute for Health Research

UNIVERSITY of York
Centre for Reviews and Dissemination

Systematic review

A list of fields that can be edited in an update can be found here

1. * Review title.

Give the title of the review in English

Analysis of different surgical approaches in the management of giant retinal tear related rhegmatogenous retinal detachment: a systematic review of literature

2. Original language title.

For reviews in languages other than English, give the title in the original language. This will be displayed with the English language title.

3. * Anticipated or actual start date.

Give the date the systematic review started or is expected to start.

01/03/2023

4. * Anticipated completion date. [1 change]

Give the date by which the review is expected to be completed.

30/04/2023

5. * Stage of review at time of this submission.

This field uses answers to initial screening questions. It cannot be edited until after registration.

Tick the boxes to show which review tasks have been started and which have been completed.

Update this field each time any amendments are made to a published record.

The review has not yet started: Yes

Review stage	Started	Completed
Preliminary searches	No	No
Piloting of the study selection process	No	No
Formal screening of search results against eligibility criteria	No	No
Data extraction	No	No
Risk of bias (quality) assessment	No	No
Data analysis	No	No

Provide any other relevant information about the stage of the review here.

6. * Named contact.

The named contact is the guarantor for the accuracy of the information in the register record. This may be any member of the review team.

Rabia Hussain

Email salutation (e.g. "Dr Smith" or "Joanne") for correspondence:

Dr. Hussain

7. * Named contact email.

Give the electronic email address of the named contact.

rabia.hussain2010@gmail.com

8. Named contact address

Give the full institutional/organisational postal address for the named contact.

Discipline of Social & Administrative Pharmacy, School of Pharmaceutical Sciences, Universiti Sains Malaysia, Pulau Pinang, 11800, Malaysia

9. Named contact phone number.

Give the telephone number for the named contact, including international dialling code.

+60172693246

10. * Organisational affiliation of the review.

Full title of the organisational affiliations for this review and website address if available. This field may be completed as 'None' if the review is not affiliated to any organisation.

Universiti Sains Malaysia, Pulau Pinang, 11800, Malaysia

Organisation web address:

http://www.usm.my/

11. * Review team members and their organisational affiliations. [1 change]

Give the personal details and the organisational affiliations of each member of the review team. Affiliation refers to groups or organisations to which review team members belong. **NOTE: email and country now MUST be entered for each person, unless you are amending a published record.**

Dr Miguel A. Quiroz-Reyes.
Dr Rabia Hussain. Universiti Sains Malaysia
Professor Zaheer-ud-Din Babar. University of Huddersfield
Miss Loh Zhe Chi. Universiti Sains Malaysia

12. * Funding sources/sponsors.

Details of the individuals, organizations, groups, companies or other legal entities who have funded or sponsored the review.

Not applicable

Grant number(s)

State the funder, grant or award number and the date of award

13. * Conflicts of interest.

List actual or perceived conflicts of interest (financial or academic).

None

14. Collaborators.

Give the name and affiliation of any individuals or organisations who are working on the review but who are not listed as review team members. **NOTE: email and country must be completed for each person, unless you are amending a published record.**

15. * Review question.

State the review question(s) clearly and precisely. It may be appropriate to break very broad questions down into a series of related more specific questions. Questions may be framed or refined using PI(E)COS or similar where relevant.

1. To analyze different surgical approaches for rhegmatogenous retinal detachment-related giant retinal tears (GRTs).

2. To provide an overview of the complication and correlated approaches with reference to best correct visual acuity (BCVA) in rhegmatogenous retinal detachment-related giant retinal tears (GRTs).

16. * Searches.
[1 change]

State the sources that will be searched (e.g. Medline). Give the search dates, and any restrictions (e.g. language or publication date). Do NOT enter the full search strategy (it may be provided as a link or attachment below.)

Sources: PubMed, Scopus, Google Scholar, Springer Link.

Articles that were published between 2001 and 2023, articles written in English language will be included.

Additional search strategy information can be found in the attached PDF document (link provided below).

17. URL to search strategy.

Upload a file with your search strategy, or an example of a search strategy for a specific database, (including the keywords) in pdf or word format. In doing so you are consenting to the file being made publicly accessible. Or provide a URL or link to the strategy. Do NOT provide links to your search **results**.

https://www.crd.york.ac.uk/PROSPEROFILES/401049_STRATEGY_20230227.pdf

Alternatively, upload your search strategy to CRD in pdf format. Please note that by doing so you are consenting to the file being made publicly accessible.

Do not make this file publicly available until the review is complete

18. * Condition or domain being studied.

Give a short description of the disease, condition or healthcare domain being studied in your systematic review.

A giant retinal tear is defined as a full-thickness retinal break of at least 90° in circumferential extent (Schepens et al., 1962; Ting et al., 2020). A rhegmatogenous retinal detachment (RRD) occurs when a tear in the retina leads to fluid accumulation with a separation of the neurosensory retina from the underlying retinal pigment epithelium. Giant retinal tears comprise about 1.5% of rhegmatogenous retinal detachments (Shunmugam, 2014). Rhegmatogenous retinal detachment associated with giant retinal tears has been treated with intentional retinal incarceration, prone air-fluid exchange and retinal tacks or sutures, but the outcome has typically been dismal. Also, another treatment option for rhegmatogenous retinal detachment secondary to giant retinal tears and potentially associated with a high anatomical reattachment rate is pars plana vitrectomy (Ghasemi et al., 2017).

19. * Participants/population.

Specify the participants or populations being studied in the review. The preferred format includes details of both inclusion and exclusion criteria.

Inclusion criteria

-The review will examine studies that include patients with retinal detachment caused by giant retinal tears (GRTs), which are defined as circumferential retinal breaks measuring at least 90 degrees.

-The inclusion criteria requires that the studies report at least one of the following outcomes: anatomical outcomes or functional outcomes.

-The surgical techniques used in the studies will include pars plana vitrectomy and pars plana vitrectomy with scleral buckle, as well as other procedures such as perfluorocarbon liquids (PFCLs) and silicone oil.

-Although the studies primarily focused on adults, those that include both adults and children will not be excluded from the review.

Exclusion criteria

-The review will exclude studies that did not report any surgical techniques, such as pars plana vitrectomy or scleral buckle, for the treatment of retinal detachment.

-Additionally, the review will exclude studies that focused on rhegmatogenous retinal detachment caused by retinal tears other than giant retinal tears.

20. * Intervention(s), exposure(s).

Give full and clear descriptions or definitions of the interventions or the exposures to be reviewed. The preferred format includes details of both inclusion and exclusion criteria.

Scleral buckle (SB); Pars plana vitrectomy (PPV); Combined PPV/SB; Combined 3-port PPV/SB; PPV with tamponade gas; PPV with silicone oil; Photocoagulation and SB; Primary TPPV without SB; Micro-incision vitrectomy surgery (MIVS).

Inclusion criteria: Original research articles which available in full text, reported rhegmatogenous retinal detachment caused by giant retinal tears and available in English language.

Exclusion criteria: Studies reported rhegmatogenous retinal detachment caused by retinal tears other than giant retinal tears, and included only pediatrics cases.

21. * Comparator(s)/control.

Where relevant, give details of the alternatives against which the intervention/exposure will be compared (e.g. another intervention or a non-exposed control group). The preferred format includes details of both inclusion and exclusion criteria.

Not applicable.

22. * Types of study to be included.

Give details of the study designs (e.g. RCT) that are eligible for inclusion in the review. The preferred format includes both inclusion and exclusion criteria. If there are no restrictions on the types of study, this should be stated.

Case series (observational case series; non-consecutive interventional case series; prospective, interventional case series study),

Retrospective studies (retrospective chart review, retrospective case series)

Cohort studies.

23. Context.

Give summary details of the setting or other relevant characteristics, which help define the inclusion or exclusion criteria.

Studies about different surgeries related to the rhegmatogenous retinal detachment secondary to giant retinal tears will be included in these studies.

24. * Main outcome(s).

Give the pre-specified main (most important) outcomes of the review, including details of how the outcome is defined and measured and when these measurement are made, if these are part of the review inclusion criteria.

- Anatomical success: refers to the successful reattachment of the retina following surgery for a retinal detachment. This means that the retina is in its proper place and is attached to the underlying tissues, as it should be. The measurement of anatomical success is based on postoperative imaging or physical examination of the retina to determine if it is properly attached.
- Functional success refers to the improvement or maintenance of visual acuity after surgical repair for retinal detachment. It is measured in terms of best corrected visual acuity (BCVA) before and after surgery, with a higher BCVA indicating a better functional outcome.

Measures of effect

Please specify the effect measure(s) for you main outcome(s) e.g. relative risks, odds ratios, risk difference, and/or 'number needed to treat.

Anatomic success: The effect measures which will be reported include single surgery anatomic success (SSAS), primary reattachment rate, and overall success rate, which will be reported as percentages.
Functional success: The effect measures reported include best-corrected visual acuity (BCVA) and logMAR. The BCVA is reported in Snellen notation, which indicates the smallest line of letters that can be read on a standard eye chart. LogMAR is a logarithmic transformation of visual acuity that is used to analyze the change in visual acuity over time. Some studies reported median visual acuity, while others reported mean or

overall visual acuity. The findings suggest that visual acuity generally improved after surgery, with a significant proportion of patients achieving good visual outcomes.

25. * Additional outcome(s).

List the pre-specified additional outcomes of the review, with a similar level of detail to that required for main outcomes. Where there are no additional outcomes please state 'None' or 'Not applicable' as appropriate to the review

None.

Measures of effect

Please specify the effect measure(s) for you additional outcome(s) e.g. relative risks, odds ratios, risk difference, and/or 'number needed to treat.

Not applicable.

[1 change]

26. * Data extraction (selection and coding).

Describe how studies will be selected for inclusion. State what data will be extracted or obtained. State how this will be done and recorded.

Articles that were published between 2001 and 2023, articles written in English language will be included. Two independent reviewers will independently judge the articles based on the inclusion criteria and the study objectives. Discrepancies were resolved through the discussion with a third-party reviewer to avoid bias. Data about anatomical success, final visual acuity and recurrent retinal attachment from each study will be extracted and written in an Excel sheet.

27. * Risk of bias (quality) assessment.

State which characteristics of the studies will be assessed and/or any formal risk of bias/quality assessment tools that will be used.

Observational case series: The Risk of Bias in Non-Randomized Studies of Interventions (ROBINS-I) tool
can be used to assess risk of bias in non-randomized studies, including observational case series.

Case report: Quality assessment of case reports can be done using the CARE Case Report guidelines.

Interventional case series: The Cochrane Risk of Bias tool can be used to assess the risk of bias in randomized and non-randomized interventional studies.

Retrospective study: The ROBINS-I tool can be used to assess risk of bias in non-randomized retrospective studies.

Non-comparable case series: The Strengthening the Reporting of Observational Studies in Epidemiology (STROBE) statement can be used to assess the quality of reporting in non-comparable case series.

Clinical study: The Cochrane Risk of Bias tool can be used to assess the risk of bias in randomized and non-

randomized clinical studies.

Consecutive series: The STROBE statement can be used to assess the quality of reporting in consecutive case series.

Case series: The STROBE statement can also be used to assess the quality of reporting in case series.

Surgical cohort: The Reporting of Studies Conducted Using Observational Routinely Collected Health Data (RECORD) statement can be used to assess the quality of reporting in surgical cohort studies.

18. * Strategy for data synthesis. [1 change]

Describe the methods you plan to use to synthesise data. This **must not be generic text** but should be **specific to your review** and describe how the proposed approach will be applied to your data. If meta-analysis is planned, describe the models to be used, methods to explore statistical heterogeneity, and software package to be used.

This study will synthesize the results based on strict inclusion criteria that is surgical management related to anatomic and functional outcomes.

Further information about inclusion criteria is given below:

(1) Studies that include patients with retinal detachment caused by giant retinal tears, which are defined as circumferential retinal breaks measuring at least 90 degrees.

(2) The inclusion criteria requires that the studies report at least one of the following outcomes: anatomical outcomes or functional outcomes.

(3) The surgical techniques used in the studies will include pars plana vitrectomy and pars plana vitrectomy with scleral buckle, as well as other procedures such as perfluorocarbon liquids (PFCLs) and silicone oil.

(4) Although the studies primarily focused on adults, those that include both adults and children will not be excluded from the review.

The data that will be synthesized include outcomes related to anatomical in terms of retinal reattachment rates and functional success in terms of best corrected visual acuity. A qualitative synthesis will be employed, as the researcher would summarise the findings of each study and identify common themes or patterns across the studies. Statistical data will be incorporated solely to indicate the scale of the findings and will not be utilized during the process of data synthesis.

The interpretation of the results will involve a qualitative analysis of the extracted data, identifying similarities

and differences between the studies. Conclusions will be drawn regarding the effectiveness of different surgical approaches for rhegmatogenous retinal detachment associated with giant retinal tears.

29. * Analysis of subgroups or subsets.

State any planned investigation of ‘subgroups’. Be clear and specific about which type of study or participant will be included in each group or covariate investigated. State the planned analytic approach.

None planned.

30. * Type and method of review.

Select the type of review, review method and health area from the lists below.

Type of review

Cost effectiveness

No

Diagnostic

No

Epidemiologic

No

Individual patient data (IPD) meta-analysis

No

Intervention

No

Living systematic review

No

Meta-analysis

No

Methodology

No

Narrative synthesis

No

Network meta-analysis

No

Pre-clinical

No

Prevention

No

Prognostic

No

Prospective meta-analysis (PMA)

No

Review of reviews

No

Service delivery

No

Synthesis of qualitative studies

No

Systematic review

Yes

Other

No

Health area of the review

Alcohol/substance misuse/abuse

No

Blood and immune system

No

Cancer

No

Cardiovascular

No

Care of the elderly

No

Child health

No

Complementary therapies

No

COVID-19

No

Crime and justice

No

Dental

No

Digestive system

No

Ear, nose and throat

No

Education

No

Endocrine and metabolic disorders

No

Eye disorders

Yes

General interest

No

Genetics

No

Health inequalities/health equity

No

Infections and infestations

No

International development

No

Mental health and behavioural conditions

No

Musculoskeletal

No

Neurological

No

Nursing

No

Obstetrics and gynaecology

No

Oral health

No

Palliative care

No

Perioperative care

No

Physiotherapy

No

Pregnancy and childbirth

No

Public health (including social determinants of health)

No

Rehabilitation

No

Respiratory disorders

No

Service delivery

No

Skin disorders

No

Social care

No

Surgery

Yes

Tropical Medicine

No

Urological

No

Wounds, injuries and accidents

No

Violence and abuse

No

31. Language.

Select each language individually to add it to the list below, use the bin icon to remove any added in error.

English

There is an English language summary.

32. * Country.

Select the country in which the review is being carried out. For multi-national collaborations select all the countries involved.

England

Malaysia

Mexico

33. Other registration details.

Name any other organisation where the systematic review title or protocol is registered (e.g. Campbell, or The Joanna Briggs Institute) together with any unique identification number assigned by them. If extracted data will be stored and made available through a repository such as the Systematic Review Data Repository (SRDR), details and a link should be included here. If none, leave blank.

34. Reference and/or URL for published protocol.

If the protocol for this review is published provide details (authors, title and journal details, preferably in Vancouver format)

Add web link to the published protocol.

Or, upload your published protocol here in pdf format. Note that the upload will be publicly accessible.

No I do not make this file publicly available until the review is complete

Please note that the information required in the PROSPERO registration form must be completed in full even if access to a protocol is given.

35. Dissemination plans.

Do you intend to publish the review on completion?

Yes

Give brief details of plans for communicating review findings.?

The findings of this review will be published in indexed journal.

36. Keywords.

Give words or phrases that best describe the review. Separate keywords with a semicolon or new line. Keywords help PROSPERO users find your review (keywords do not appear in the public record but are included in searches). Be as specific and precise as possible. Avoid acronyms and abbreviations unless these are in wide use.

Rhegmatogenous retinal detachment; Giant retinal tears; Anatomical success; Functional success

37. Details of any existing review of the same topic by the same authors.

If you are registering an update of an existing review give details of the earlier versions and include a full bibliographic reference, if available.

38. * Current review status.

Update review status when the review is completed and when it is published.New registrations must be

ongoing so this field is not editable for initial submission.

Please provide anticipated publication date

Review_Ongoing

39. Any additional information.

Provide any other information relevant to the registration of this review.

This review is planned to analyze different surgical techniques based on the functional and anatomic success to treat rhegmatogeneous detachment due to giant retinal tears.

40. Details of final report/publication(s) or preprints if available.

Leave empty until publication details are available OR you have a link to a preprint (NOTE: this field is not editable for initial submission). List authors, title and journal details preferably in Vancouver format.

Give the link to the published review or preprint.
