# Supplementary material for: Management, risk factors and treatment outcomes of rhegmatogenous retinal detachment associated with giant retinal tears: scoping review
Source: Int J Retina Vitreous. 2024 Apr 23;10:35. doi: 10.1186/s40942-024-00552-6 (PMC11036595; doi:10.1186/s40942-024-00552-6)
Supplement: Supplementary file 2 — Supplementary Material 2 [file 40942_2024_552_MOESM2_ESM.docx]

**Supplementary file. Appendix 1**

**Table S1: Details about databases searched and search strategy**

| **Databases** | **Search strategy** |
| --- | --- |
| **PubMed** | ((((((("giant s"[All Fields] OR "gigantism"[MeSH Terms] OR "gigantism"[All Fields] OR "giant"[All Fields] OR "giants"[All Fields]) AND ("retinal perforations"[MeSH Terms] OR ("retinal"[All Fields] AND "perforations"[All Fields]) OR "retinal perforations"[All Fields] OR ("retinal"[All Fields] AND "tears"[All Fields]) OR "retinal tears"[All Fields])) OR "GRT"[All Fields]) AND (("rhegmatogeneous"[All Fields] OR "rhegmatogenic"[All Fields] OR "rhegmatogenous"[All Fields]) AND ("retinal detachment"[MeSH Terms] OR ("retinal"[All Fields] AND "detachment"[All Fields]) OR "retinal detachment"[All Fields]))) OR "RRD"[All Fields]) AND (("surgical procedures, operative"[MeSH Terms] OR ("surgical"[All Fields] AND "procedures"[All Fields] AND "operative"[All Fields]) OR "operative surgical procedures"[All Fields] OR "surgical"[All Fields] OR "surgically"[All Fields] OR "surgicals"[All Fields]) AND ("approach"[All Fields] OR "approach s"[All Fields] OR "approachability"[All Fields] OR "approachable"[All Fields] OR "approache"[All Fields] OR "approached"[All Fields] OR "approaches"[All Fields] OR "approaching"[All Fields] OR "approachs"[All Fields]))) OR ("complicances"[All Fields] OR "complicate"[All Fields] OR "complicated"[All Fields] OR "complicates"[All Fields] OR "complicating"[All Fields] OR "complication"[All Fields] OR "complication s"[All Fields] OR "complications"[MeSH Subheading] OR "complications"[All Fields]) OR ("pars"[All Fields] AND "plana"[All Fields] AND ("vitrectomy"[MeSH Terms] OR "vitrectomy"[All Fields] OR "vitrectomies"[All Fields])) OR "PPV"[All Fields] OR ("scleral buckling"[MeSH Terms] OR ("scleral"[All Fields] AND "buckling"[All Fields]) OR "scleral buckling"[All Fields]) OR ("stat bull metrop insur co"[Journal] OR "nat struct biol"[Journal] OR "sb"[All Fields]) OR ("tamponade"[All Fields] OR "tamponaded"[All Fields] OR "tamponades"[All Fields] OR "tamponading"[All Fields]) OR ("gas"[All Fields] OR "gasoline"[MeSH Terms] OR "gasoline"[All Fields] OR "gasolines"[All Fields] OR "petrol"[All Fields] OR "petroleum"[MeSH Terms] OR "petroleum"[All Fields] OR "petroleums"[All Fields]) OR ("silicon"[MeSH Terms] OR "silicon"[All Fields] OR "silicon s"[All Fields] OR "silicons"[All Fields]) OR ("best"[All Fields] AND ("visual acuity"[MeSH Terms] OR ("visual"[All Fields] AND "acuity"[All Fields]) OR "visual acuity"[All Fields])) OR "BVA"[All Fields]) AND ((ffrft[Filter]) AND (clinicaltrial[Filter] OR randomizedcontrolledtrial[Filter]) AND (fft[Filter])) Filters: Free full text, Full text, Clinical Trial, Randomized Controlled Trial, English, Exclude preprints, from 2001/1/1 - 2023/3/20 |
| **Scopus** | giant  AND  retinal  AND  tears  OR  grt  AND  rhegmatogenous  AND  retinal  AND  detachment  OR  rrd  AND  surgical  AND  approaches  OR  complications  OR  pars  AND  plana  AND  vitrectomy  OR  ppv  OR  scleral  AND  buckling  OR  sb  OR  tamponade  OR  gas  OR  silicon  OR  best  AND  visual  AND  acuity  OR  bva  AND  ( LIMIT-TO ( DOCTYPE ,  "ar" )  OR  LIMIT-TO ( DOCTYPE ,  "cp" ) )  AND  ( LIMIT-TO ( LANGUAGE ,  "English" )  OR  LIMIT-TO ( LANGUAGE ,  "Turkish" )  OR  LIMIT-TO ( LANGUAGE ,  "Chinese" )  OR  LIMIT-TO ( LANGUAGE ,  "Czech" )  OR  LIMIT-TO ( LANGUAGE ,  "Japanese" )  OR  LIMIT-TO ( LANGUAGE ,  "Russian" ) )  AND  ( LIMIT-TO ( PUBYEAR ,  2023 )  OR  LIMIT-TO ( PUBYEAR ,  2022 )  OR  LIMIT-TO ( PUBYEAR ,  2021 )  OR  LIMIT-TO ( PUBYEAR ,  2020 )  OR  LIMIT-TO ( PUBYEAR ,  2019 )  OR  LIMIT-TO ( PUBYEAR ,  2018 )  OR  LIMIT-TO ( PUBYEAR ,  2017 )  OR  LIMIT-TO ( PUBYEAR ,  2016 )  OR  LIMIT-TO ( PUBYEAR ,  2015 )  OR  LIMIT-TO ( PUBYEAR ,  2014 )  OR  LIMIT-TO ( PUBYEAR ,  2013 )  OR  LIMIT-TO ( PUBYEAR ,  2012 )  OR  LIMIT-TO ( PUBYEAR ,  2011 )  OR  LIMIT-TO ( PUBYEAR ,  2010 )  OR  LIMIT-TO ( PUBYEAR ,  2009 )  OR  LIMIT-TO ( PUBYEAR ,  2008 )  OR  LIMIT-TO ( PUBYEAR ,  2007 )  OR  LIMIT-TO ( PUBYEAR ,  2006 )  OR  LIMIT-TO ( PUBYEAR ,  2005 )  OR  LIMIT-TO ( PUBYEAR ,  2004 )  OR  LIMIT-TO ( PUBYEAR ,  2003 )  OR  LIMIT-TO ( PUBYEAR ,  2002 )  OR  LIMIT-TO ( PUBYEAR ,  2001 ) )  AND  ( EXCLUDE ( LANGUAGE ,  "Turkish" )  OR  EXCLUDE ( LANGUAGE ,  "Chinese" )  OR  EXCLUDE ( LANGUAGE ,  "Czech" )  OR  EXCLUDE ( LANGUAGE ,  "Japanese" )  OR  EXCLUDE ( LANGUAGE ,  "Russian" ) ) |
| **Google scholar** | Giant retinal tears OR GRT AND rhegmatogenous retinal detachment OR RRD AND surgical approaches OR complications OR pars plana vitrectomy OR PPV OR scleral buckling OR SB OR tamponade OR gas OR silicon OR best visual acuity OR BVA |
| **Springer Link** | Giant retinal tears OR GRT AND rhegmatogenous retinal detachment OR RRD AND surgical approaches OR complications OR pars plana vitrectomy OR PPV OR scleral buckling OR SB OR tamponade OR gas OR silicon OR best visual acuity OR BVA |

**Table S2.** Characteristics of Study such as Country, Study Design, Setting, Inclusion Criteria, Exclusion Criteria, Surgical Repair, Comparison, Presence of Giant Retinal Tears, Postoperative Follow-up Period, Number of Patients, and Number of Eyes

| **Reference** | **Country** | **Study design** | **Study setting** | **Inclusion criteria** | **Exclusion criteria** | **Surgical repair** | **Comparisons** | **Presence of GRT** | **Postoperative follow-up period** | **Number of patients** | **Number of eyes** |
| --- | --- | --- | --- | --- | --- | --- | --- | --- | --- | --- | --- |
| Sung *et al*., 2020 [36] | South Korea | A retrospective cohort study | Ophthalmology, Chungnam National University  Hospital. | This study enrolled patients who had undergone surgery for RRD and had a follow-up period of at least six months after the surgery, based on the review of medical records from January 2007 to June 2016. | The study excluded patients who had undergone ocular surgery other than simple cataract surgery and those with other ocular diseases such as tractional or exudative RD, macular degeneration, and retinal vein occlusion. | Combined PPV with SB were done for patients who had total RRD with preoperative PVR, trauma, or multiple retinal tears. Either a sponge or band was used for combined SB and PPV. The PPV surgery was done using a 23-g instrument. In the total RRD group, PPV alone was conducted in 38 (86.4%) eyes, while combined PPV and SB were conducted in 6 (13.6%) eyes. In the total RRD group, gas (SF_6_, C_3_F_8_) tamponade was used in 23 (52.3%) eyes, while SO tamponade was used in 21 (47.7%) eyes. Data on the number of surgeries conducted for partial RRD group was not available. | 88 patients with partial RRD (control group): 44 patients with total RRD. | In the total RRD group, six out of 44 eyes have GRT. In the partial RRD group, two out of 88 eyes have GRT. 34.8% of the total 132 eyes were presented with GRT. | Mean = 28.61 months | 44 patients with total RRD whereas 88 patients with partial RRD | 44 eyes with total RRD whereas 88 eyes with partial RRD |
| Li *et al*., 2021 [10] | The United States of America | A retrospective, non-consecutive interventional case series | the University of Michigan W.K. Kellogg Eye Center | Between January 2011 and July 2020, this study included patients, including children, who were diagnosed with GRT-related RRDs and underwent primary surgical repair with PPV, SB, or PPV/SB. The patients also included those with a history of trauma such as open globe injury, hereditary vitreoretinopathies, or grade C PVR. | Patients who have a follow-up period of less than 90 days. | 83% involved only PPV, while 15% of the eyes had a combined PPV/SB surgery, and only 2% had a primary SB surgery. Only one eye with a superior GRT received an SB, while seven eyes with inferior GRTs received SBs. The surgeries were performed using either 23-g (38%) or 25-g (62%) instrumentation, and most of the surgeries involved the use of PFCL  (90%). C_3_F_8_ was the most commonly used internal tamponade (81%), and SO was used in only 19% of cases after a fluid-air exchange. | Not applicable. | All the included eyes. The extent of the GRT was <180° in 35 eyes (73%) and located inferiorly in 29 eyes (60%) | Median = 28 months | 47 | 48 |
| Oderinlo *et al*., 2020 [37] | Africa | A retrospective non-comparable case series | Eye Foundation Hospital Retina Institute | Patients  undergoing primary RD repair for RRD between January 2014 and December 2018. | History of retinopathy of prematurity, tractional RD from diabetic retinopathy, sickle cell retinopathy, choroidal exudative RDs, and RDs from posterior uveitis were excluded from this study | Four different types of surgical interventions were done, combined three port PPV with SO exchange and SB was done in 12 eyes (14.8%) in group A and 33 eyes (17.0%) in group B, only SB with or without drainage of subretinal fluid was done in no group A eye and 15 eyes (7.7%) in group B, PPV with SO exchange was done in 68 eyes (84%) in group A and 138 eyes (71.1%) in group B, PPV with gas for  tamponade gas was done in one eye (1.2%) in group A and 8 eyes (4.1%) in group B. Surgery was done using 23-g or 25-g instruments, PFCLs were used at the surgeons’  discretion for both GRT and other holes or tears. | To compare the outcomes of RRD surgeries between the group with GRT and other types of tears. Group A (N=7, 9.7%) is the GRT group, while group B (N =27, 14.8%) is other holes group | 81 eyes (29.4%) | Not stated | 275 | 275 |
| Ting *et al*., 2020 [38] | Singapore | A retrospective cohort study | Singapore National Eye Centre | All patients with GRT-related RRD | Not stated | 33.1% eyes underwent PPV from 1991-2015, in which the number of cases during 1991-2005 recorded a higher number of cases as compared to 2006-2015, corresponding to 28 (35.9%) eyes and 14 (28.6%) eyes respectively. On the other hand, 85 (66.9%) eyes underwent combined PPV and SB, 50 (64.1%) eyes for the period of 1991-2005, and 35 (71.4%) eyes recorded for the period of 2006-2015. PFCL had been used in  102 (80.3%) eyes, the number of cases increased from 73.1% (1991-2005) to 91.8% (2006-2015). For the endotamponade, 30 (23.6%) eyes used SF6, 24 (18.9%) eyes used C_2_F_6_, 41 (32.3%) eyes used C3F8, and 32 (25.2%) eyes used SO. | To compare the functional and anatomical outcome of GRT-related RRD surgical approaches that were conducted between the year of 1991-2015 (Group A, n=127);1991–2005 (Group B, n=78)  and 2006–2015 (Group C, n=49) | All the included eyes | A minimum of five years | 127 | 127 |
| Mikhail *et al*., 2017 [39] | The United Kingdom | A retrospective case series | Vitreoretinal service, Royal Victoria Hospital, Belfast, Northern Ireland. | Patients who underwent surgeries for primary RRD between January 2013 and December 2013. | Cases of other types of retinal detachment or cases with  previous history of vitreoretinal procedures was excluded. | 82.5% (175) of the cases had PPV, 12.5% (27) had SB and 5% (10) had PR as a primary procedure. Of those who had PPV as a primary procedure, 67% were operated by classic 20-g PPV and the remainder by 23-g transconjunctival sutureless technique. Two patients had combined cataract and PPV. SBs were circumferential in 55%, segmental in 38% and radial in 7%; 28% had subretinal fluid drainage with buckling. PR as a primary procedure was only used in uncomplicated phakic detachments. | Not applicable | Three patients (1.4%) | Mean = nine months | 211 | 212 |
| Ghasemi *et al*., 2017 [40] | Iran | A retrospective non-comparative case series | Rassoul Akram Hospital | All the patients with the diagnosis of RRD associated with GRT and underwent surgery with at least three months of follow up from 2005 to 2015. | Concomitant presence of any other ocular pathology which could decrease VA such as diabetic retinopathy, advanced glaucoma, and previous uveitis at presentation. | PPV alone was performed in 44 eyes (71.0%) and simultaneous PPV and phacoemulsification surgery was performed in 18 eyes (29.0%). An encircling SB was placed in seven eyes (11.3%). All eyes received SO tamponade and had 360-degree laser photocoagulation of the peripheral retina, intraoperatively. Heavy SO was injected in 10 (16.1%) eyes. | Not applicable | All the included eyes, while the GRT was 180° or greater in 20 (32.2%) eyes and less than 180° in 42 (67.8%) eyes. | Mean = 21.54 ± 27.73 (three –126) months | 61 | 62 |
| Lumi *et al*., 2016 [41] | Slovenia | A retrospective cohort study | Eye Hospital, University Medical Centre Ljubljana, Slovenia | Patients with subtotal or total RRD with a GRT, retinal dialysis, multiple retinal breaks, posterior breaks, RRD with vitreous hemorrhage, RRD after penetrating eye injury and RDs with preoperative PVR grade C1 or higher, who underwent vitrectomy were included in the study | Patients younger than 16 years old, aphakic, having PDR or retinal dystrophies and three patients with incomplete follow-up period | In all cases, 23- or 25-g PPV was performed. PPV alone was performed in 117 cases. 89 (76.1%) eyes using (non-expansile C_3_F_8_ tamponade as gas tamponade and 28 (23.9%) eyes using SO tamponade. PPV with phacoemulsification and IOL implantation was only performed in three cases with dense cataract. | To compare the surgical outcomes in different groups. Group one (n = 46): PPV in phakic eyes + gas  Tamponade  Group two (n = 43): PPV in pseudophakic eyes + gas  Tamponade  Group three (n = 13): PPV in phakic eyes + SO  Tamponade  Group four (n = 15): PPV in pseudophakic eyes + SO  tamponade.  The second classification was done according to the lens status and the presence or absence of preoperative PVR grade C1 or more:  Group five (n = 43): PPV in phakic eye without preoperative PVR C1  Group six (n = 48): PPV in pseudophakic eye without preoperative PVR C1  Group seven (n = 16): PPV in phakic eye with preoperative PVR ≥ C1  Group eight (n = 10): PPV in pseudophakic eye with preoperative PVR ≥ C1  Additionally, the eyes were classified according to their AL (n = 93):  Group nine (n = 40): PPV in eyes with AL  ≤ 24 mm  Group 10 (n = 53): PPV in eyes with AL > 24 mm | Yes, but the number of cases were not stated. | At least 12 months post-operatively. | 117 | 117 |
| Barth *et al*., 2023 [42] | Germany | A retrospective cohort study | Data was obtained from patients' surgical logbooks | Patients with good preoperative Snellen's VA of ≥ 0.3, RRD with macula on status and SO as primary tamponade and at least three months of follow-up after SO removal | Patients with recurrent RRD after primary PPV, PVR grade C or higher, history of trauma or previous intraocular surgery, glaucomatous optic disc changes or other pre-existing ocular diseases affecting visual outcome as well as postoperative complications such as endophthalmitis, ERM or outer retinal layer defects, distinct macular edema, or subretinal PFCL remnants | All 22 eyes had been primarily treated with a standard 20-g (until 2010) or a 23-g PPV (from 2011 onwards) with SO tamponade. After vitrectomy and drainage of subretinal fluid with PFCL, retinal breaks and tears were treated with cryo- or laser-photocoagulation, or both. Afterwards, PFCL was removed completely and exchanged first for air and afterwards air for SO. | To compare the surgical outcomes of patients with unexplained visual loss during or after SO tamponade for macula on RD repair were compared to patients with good functional outcome. | 20 eyes; 91% | Mean = 20 months (SD 30.6) after SO removal | Not stated | 22 |
| Murtagh *et al*., 2020 [43] | Ireland | A retrospective cohort study | The Mater  Misericordiae University Hospital and the Mater Private  Hospital | A diagnosis of primary  RRD in patients aged 16 years  and over. | Previous RD  surgery, tractional or exudative RD and patients less than 16 years old. | Over the four years recorded, 331 cases out of the 613 (54.00%) cases of PPV alone were performed. PPV-SB consisted of 153 (24.96%) cases and SB with the remaining 129 (21.04%) surgeries.  The type of tamponade used in the vitrectomy surgery included 207 (42.77%) C_2_F_6_, 97 (20.04%)  C_3_F_8_, 58 (11.98%) SF_6_, 99 (20.45%) used SO. 51 used 1000 cSt,  11 had 5000 cSt inserted, nine had heavy SO, 28 had an undefined type of SO injected and air was used in  23 (4.75%) of the surgeries. In 245 (50.62%) of the vitrectomies, 23-g  sclerostomies were used, while the remaining 239 (49.38%) cases utilized 20-g sclerostomies. | Not stated | 22 eyes | Six months | 602 | 613 |
| Al-Wadani *et al*., 2014 [44] | Saudi Arabia | A retrospective case series | Department of Ophthalmology at San Gerardo Hospital, Monza, Italy | Patients who underwent SO removal between 2000 and 2010. | Not stated | All eyes underwent prophylactic  360º laser retinopexy and 360º SB at the  time of primary surgery. Fluid-SO exchange  was performed using an automated pump. In all patients, SO removal was performed  through a pars plana incision  using a 19-g needle connected to a vacuum pump | Not stated | 13 eyes (7.1%) | 66.9 weeks after SO removal | 177 | 184 |
| Zanzottera *et al*., 2022 [45] | Italy | A retrospective case series | Department of Ophthalmology at San Gerardo Hospital, Monza, Italy | All the consecutive patients with complicated RD underwent 25- or 23-g PPV and DF. between December 2018 and February 2021. DF consists of the use of both PFCL and SO to temporarily stabilize the retina before exchanging with SO. Both types of PFCL used in this study were (C_10_F_18_) and PDMS. | Eyes missing detailed data or achieving good anatomical results with less than three months follow up, tractional RDs in proliferative diabetic retinopathy were excluded. | All patients underwent small gauge PPV, with 13 (86.7%) eyes received 25-g PPV, two (13.3%) eyes 23-g PPV, internal tamponade with C_10_F_18_ (60% of vitreous cavity) and PDMS (40% of vitreous cavity), endolaser photocoagulation, and postoperative supine positioning until PFCL removal. | Not applicable | Six out of 15 eyes (40%) | Median = six months (range one –22, mean = 9,4 months) | 15 | 15 |
| Bai *et al*., 2022 [46] | China | A retrospective case series | Qilu Hospital, Shandong University and other four hospitals included one tertiary general hospital, one hospital focused on ophthalmology and two were local general hospitals | Two kinds of case series between 2012 to 2020 were presented in this study. The first case series involved patients with RDs, who were treated by vitrectomy and the use of PFCLs, while the second case series included patients who were treated by VWTPL. | The second case series excluded the cases in which PFCL was used to flatten the retina intraoperatively if the flattening was failed by using air-fluid exchange even after retinotomy. | PPVs were conducted in all the cases by complete removal of proliferative membrane and posterior vitreous membrane (with the aid of triamcinolone acetonide), air-fluid exchange, in situ photocoagulation and SO tamponade (when necessary) | Not stated | 20 eyes (6.3%) | Range = One -26 months | 320 | 320 |
| Quiroz-Reyes *et al*., 2022 [47] | Mexico | A retrospective, consecutive, multicenter cohort study | Retina  Specialists Unit at Oftalmologia Integral ABC in  Mexico City | Only the charts of patients aged ≥18 years had GRT-associated with RRD, evidence of PVR grade B or less, retina attached at the last follow-up examination visit, postoperative BCVA in the functional range (20/800 or better), absence of intraocular SO in the last follow-up visit, at least six months of follow-up, and a well-serial documented structural and functional assessment of the macula during follow-up were included. | Patients were excluded from the study if they had a history of complicated vitreoretinal surgery or intravitreal injections, GRT-related RRD due to penetrating or perforating open-eye injury, GRT-related RRD combined with MHRD due to myopic traction maculopathy, postoperative BCVA of 20/800 or worse, presence of intraocular SO at the last follow-up evaluation visit, severe grade C posterior PVR or anterior PVR with evidence of recurrent, complicated RRD at the last follow-up visit, history of active glaucoma, impossibility to follow-up, loss of follow-up, surgery in a non-designated institution, presence of severe complications such as endophthalmitis, recurrent, complicated severe PVR RRD at the last follow-up visit evaluation, and refractory corneal opacity development during follow-up. | A standard 23- or 25-g PPV was performed in all the eyes. The retina was reattached by a PFCL-assisted technique to effectively perform hydropneumatics retinal manipulation and assisted subretinal fluid expression to completely dry out the subretinal space, a second air-fluid exchange was performed, and as the last surgical step, a non-expandable bubble containing 15% C_3_F_8_ gas mixture or lighter than water SO was used as a long-acting tamponade at the end of the procedures in all cases. | To compare anatomical and functional outcomes in successfully operated eyes with analysis of the subset depending upon the degree of GRT-associated RRD extension classified as follows: group 1 (n = 42 eyes) with GRT-associated RRD extension < 180°; group 2 (n = 23 eyes) with GRT-associated RRD extension = 180°-270°; and group 3 (n = 11 eyes) with GRT-associated RRD extension > 270°. | 76 eyes presented with GRT. Of the 76 eyes, 42 eyes associated with circumferential retinal tears <180° (group 1), 23 eyes had RRD associated with circumferential retinal tears between 180 °and 270° (group 2), and 11 eyes had RRD associated with circumferential GRTs >270° (group 3). | Mean = 28.1 months | 66 | 76 |
| Oderinlo *et al*., 2012 [48] | Nigeria | A retrospective non-comparative interventional case series | Eye  Foundation Hospital, Lagos | Patients who had surgery for RRD between June 2006 and December 2007. | Not stated | Four different types of surgical interventions were done. Combined 20-g PPV with SO exchange and SB was done in 11eyes (10.7%) while only SB with or without the drainage of subretinal fluid was done in 14 (13.6%) eyes, 77 eyes (74.8%) had 3PPV with SO exchange, while one eye (0.9%) had PPV with C_3_F_8_ gas for tamponade. | Not applicable | Nine eyes (8.7%) | Range = Three -24 months,  mean = 8.3 ± 5.9 months. | 103 | 103 |
| Ambiya *et al*., 2018 [49] | India | A retrospective cohort study | L. V. Prasad Eye Institute,  Hyderabad | Patients  ≥18 years; recurrent RD following PPV with or  without encircling band for RRD; etiologies for RRD being primary rhegmatogenous, trauma, GRT, high myopia, and cataract/cornea/glaucoma-related surgical  procedures. | Patients were excluded if their age was  <18 years, RD was tractional, exudative, combined,  post-scleral-buckle or secondary to  endophthalmitis | PPV with or without encircling band | Not applicable | Six (4.51%) eyes | Mean ± SD = 20.06 ± 12.54months | Not stated | 133 |
| Tabandeh *et al*., 2019 [50] | The United States of America | A retrospective, consecutive case series | Good Samaritan  Hospital, Los Angeles, California, USA | Patients who underwent PPV for the management  of RRD between January 2007 and September 2016, including RD associated with high myopia, GRT and failed  previous RD surgeries. | Patients younger  than 18 years, eyes with PDR, RD associated with open globe injury and cases with follow-up period less than three months were  excluded from the study. | The surgical technique included 27-g PPV (10), 25-g PPV (103) and 23-g PPV (199). Adjunct SB procedure was  performed in 106 (34%) eyes. Tamponade agents included SF_6_ gas in 16 (5%) eyes, C_3_F_8_ gas in 206 (66%) eyes, SO in 89 (29%) eyes and air in one eye | Not applicable | 14 eyes (5%) | Mean = 23.1 months | 302 | 312 |
| Ali TR. 2014 [51] | Bangladesh | A prospective cohort study | Not stated | Cases with at least six months of follow-up, retinal breaks comprised any of the following:  i) A GRT (a break ≥ three retinal clock hours)  ii) Breaks of any size or number, so long as at  least one is in the inferior retina  (between the five- and seven-o’clock position), ≥ two breaks of any size (if not in the inferior retina) located at least ≥three retinal clock hours away from one another (breaks in multiple retinal quadrants), underwent PPV with postoperative PFO retention was performed as the primary (initial) retinal reattachment procedure. | The cases with RRD that were repaired by PPV with postoperative PFO were excluded from the study if they met any of the following criteria: (1) had macular disease before the retinal detachment was clinically evident or had been previously documented; (2) involved endophthalmitis or a penetrating eye injury; (3) had a previous vitrectomy performed in the study eye for any indication. | All the eyes underwent a standard three-port 20-g vitrectomy with scleral buckling procedure. In 43 patients (Group one) PFO was exchanged with SO in the same surgical procedure and in 22 patients (Group two) PFO was kept for three days and then exchanged with silicon oil by a separate surgical  procedure. | To compare between group one, n=43, in which PFO was exchanged with silicon  oil in the same surgical procedure and group two, n = 22, in which PFO was kept for three days and then exchanged with SO by a separate surgical  procedure. | Yes, but the exact numbers are not stated. | Range = six -19 months | 65 | 65 |
| Haugstad *et al*., 2017 [52] | Norway | A retrospective cohort study | Department of Ophthalmology, Oslo University Hospital (OUH), Oslo | A clinical diagnosis of primary RRD including severe PVR grade B and C. | Previous vitreoretinal surgery and penetrating ocular trauma | PPV in 317 eyes (61.3%), PPV-SB in 23 eyes (4.5%), SB in 175 eyes (33.9%) and PR in two eyes (0.4%). In the PPV cases, 93.4% used Gas (SF_6_, C_3_F_8_, C_2_F_6_) tamponade, 2.2% employed air tamponade, 4.4% used SO tamponade. For PPV-SB cases, 82.6% used gas (SF_6_, C_3_F_8_, C_2_F_6_) tamponade, 17.4% used SO tamponade. In SB cases, 11.4% used gas tamponade, 2.3% used air tamponade, while 86.3% used no tamponade. In PR cases, 100% employed gas (SF_6_, C_3_F_8_, C_2_F_6_) tamponade | Not applicable | n=11 (2.1%) | Six months | 514 | 517 |
| Scheerlinck *et al*., 2018 [53] | Netherlands | A prospective, observational cohort study | University Medical Center Utrecht, a tertiary referral center | Patients with a RRD who were scheduled for vitrectomy with gas or SO tamponade were eligible, and consecutive patients were included to form four different groups of 10 patients each: macula on with gas; macula on with SO; macula off with gas; and macula-off with SO | Pre-existing diseases affecting the macula or the optic nerve, redetachment with macular involvement and age <18 years, structural abnormalities on the postoperative OCT-scan that possibly affected visual acuity (e.g., macular edema, subfoveal fluid, ERM and with postoperative cataract that affected visual acuity | 20- or 25-g PPV were conducted for all the patients. Air, SF_6_ or C_3_F_8_ were used for gas tamponade in 20 eyes whereas Siluron® 2000 was the SO tamponade used in another 20 eyes. | To compare the use of gas tamponade and SO tamponade in macula-on and macula-off eyes | Three eyes with macula-on RRD, while one eye with macula-off RRD | Two months after the primary vitrectomy | 40 | 40 |
| Shu *et al*., 2019 [54] | Japan | A multicenter, retrospective cohort study | Hyogo College of Medicine, Tsukuba University, Kagoshima University, Mie University, Sapporo City General Hospital, Tokushima University, Fukui University, Tokyo Medical University and the National Defense Medical College | RRD patients <50 years of age with peripheral tear | Patients with a history of eye surgeries, traumatic RRD, MHRD, retinopathy of prematurity and/or PVR. | All patients underwent surgical procedures at the discretion of retinal surgeons in each hospital. SB was performed by silicone SB with cryopexy; PPV was performed at 25- or 27-g. The selected surgeries were SB for 295 eyes (49.7%) and PPV for 262 eyes (44.1%). Patients in the combined PPV and SB group were excluded from further analysis. Tamponade materials were air, SF_6_, C_3_F_8_ and SO. | Not stated | Yes, but the exact number of cases were not stated | Not stated | 562 | 594 |
| Eiger-Moscovich *et al*., 2017 [55] | Israel | A retrospective case series | A tertiary university-affiliated medical center | Patients who were presented with GRT, treated by PPV with short-term PFCL tamponade from 2011 to 2015. Only patients who were followed for three months or more were included. | Not stated | All the patients were treated by PPV, either 25- or 23-g instruments were used, the retina was reattached using intravitreal infusion  of PFCL followed by 360º laser photocoagulation. The PFCL remained in the vitreous cavity for approximately 10 days, unless otherwise indicated. It was then extracted and replaced. PFCL was replaced by SF_6_  gas in nine (69.2%) patients, C_3_F_8_ gas in one (7.7%) patient, and SO in one (7.7%) patient. | Not applicable | All the 13 eyes | Range = three to 44 months (mean ± SD 11 ± 11 months) | 13 | 13 |
| Adelman *et al*., 2013 [70] | 48 countries | Non-randomized, multicenter, retrospective cohort study. | Members of the members of the EVRS to report on the RRDs, they operated from April 2010 to April 2011. | Due to many different RDs mentioned by the authors, only the evaluation of the treatment of RDs  with large or GRT were focused on this systematic review. The inclusion criteria were treatments of RDs with large or GRT. | Only cases with choroidal detachment  or hypotony were excluded. | All the eyes were treated with either vitrectomy or SB, with either gas or SO tamponades were used. | To compare the failure rates of vitrectomy alone and SB alone. | 1167 eyes (15.2%) | Range = three -12 months | Not stated | 7678 (all RRD cases) |
| Li *et al*., 2020 [56] | China | A retrospective case series | Beijing Tongren Eye Center | All patients who presented with primary RRD, underwent 25-g PPV  with air tamponade from August 2016 to May 2018 were included in the study. | Not stated | All the eyes were treated by standard 25-g PPV. After complete release of  traction on retinal tears, the retina was flattened by either injection of PFCL or fluid–air exchange and the subretinal fluid was drained out through the primary breaks in all cases. After fluid–air exchange, air was left in the vitreous cavity as internal tamponade. | Not stated | Three eyes (5.1%) | Mean = 12.90 ± 5.92 months (Range = 6.07–26.10 months | 59 | 59 |
| Abdala-Caballero *et al*., 2022 [57] | Colombia | A retrospective case series | A referral center in the city of Barranquilla, Colombia | Patients of legal age, diagnosed  with GRT-related RRD, treated between January 2009 and December 2018, and have a minimum of one year postoperative follow- up were included in the study. | Patients with a history of PVR grade D1 or  higher, PDR, inflammatory  diseases, and inferior and/or temporal dialysis  presence was excluded from the study. | All eyes underwent 23-g PPV with SB  and SO as tamponade. The mobile retina was flattened and stabilized by injection of PFCL. | Not applicable | All the 32 eyes. During surgery, the size of GRT was assessed:  ≤ 90° in 10 patients (32%), > 0° and < 180°  in 17 patients (52%), and ≥ 180° in five (16%). | Mean = eight months, range six –36 months | 32 | 32 |
| Ghosh *et al*., 2004 [58] | The United Kingdom | A retrospective case series | Birmingham and Midland Eye Centre. | These patients underwent surgery to repair RRD due to GRT between November 1991 and June 2002. | Not stated | All the patients underwent a PPV. PFCL was used to flatten the retina intraoperatively in 23 patients (79.31%) and six (20.68%) had air tamponade. SO tamponade was used in 28 cases (96.55%). C_3_F_8_-16% gas was used in one case. In all, 16 (55.17%) patients had explants, which included 12 encircling SB, one  segmental, and three being a combination of both. | Not stated | A total of 20 eyes (69.0%) had GRT that extended between three and six O' clock hours circumferentially, with two eyes having 360° tears. In 16 (55.17%) of these cases, GRTs occupied a predominantly/partially superior part of the retina. The other 13 occupied the inferior retina either wholly or partially. | Postoperative follow-up period ranged from five months to seven years (mean 28 months) | 29 | 29 |
| Kumar *et al*., 2018 [59] | India. | A retrospective case series | A tertiary eye care center in North India | All patients with GRT-associated (irrespective of etiology) RD who underwent 25-gauge PPV were included in the study. The  study period ranged from January 2015 to May 2016. | Patients with history of penetrating trauma, previous vitreoretinal surgery, and final follow-up less than three months. The eyes that had undergone vitrectomy before the study period with other gauges (23/20) were not included. | All patients underwent 25-gauge PPV using  a sutureless transconjunctival approach. 41.1%, PFCL is used in 82.3% of cases. Endotamponade with SF_6_ (23.6%) or SO (76.4%). | Not applicable. | All the 17 cases. | Mean = 10.2 months, range =  seven to 16 months. | 17 | 17 |
| Schwartz *et al*., 2002 [60] | The United States of America | A retrospective, non-consecutive case series | McPherson Retina Centre, Baylor College of Medicine | Patients with primary RRD, treated with SB by the McPherson Associates, for which the 20 years of follow-up were available | Eyes with PVR grade C or greater, or with a history of SB, vitrectomy, posterior segment open-globe trauma, or significant concurrent eye disease which could compromise postoperative VA such as amblyopia, macula-disease etc., were excluded | 93% of eyes (n=210) were treated with a SB. The encircling elements was a rod in 94 eyes (45%), a band with 83 eyes (40%), and could not be determined in 33 eyes (16%). | Not applicable | Four eyes (1.8%) | 20 years | 212 | 227 |
| Filippelli *et al*., 2021 [61] | Italy | A retrospective, case series | University of Molise, Campobasso, Italy | Patients treated for fovea-off RRD with PPV and SO injection between December 2018 and June 2020. | Patients were excluded if they had a previous history of ophthalmic surgery (except for noncomplicated phacoemulsification), degenerative myopia (defined as the AL of the globe ≥26.5 mm associated with degenerative changes in the sclera, choroid, and retinal pigment epithelium/retina, glaucoma, wet age-related macular degeneration, ocular vascular diseases such as diabetic retinopathy and retinal vein occlusion, and gross macular ERM. | 23-g PPV was performed in all cases. PFCL was used at the discretion of the surgeon. 1000-cSt SO was used as the final tamponade in all cases. All the eyes underwent a second surgical procedure to remove SO, two to five months after the primary surgery. | Not stated | Three eyes (6.8%) | Mean = 4.8 ± 2.7 months (range Three – nine months) | 44 | 44 |
| Christensen *et al*., 2012 [62] | Denmark | A retrospective case series | An institution database search | Eyes that were found underwent primary surgery for RRD from 2004 to 2005. | The study excluded eyes with undocumented macula-on detachments, VA of less than 6/12 before surgery, those that required repeat surgery for RRD, those with incomplete retinal attachment at last follow-up, phakic eyes that were not later operated for cataract, and eyes where the SO remained in the eye. | All patients had undergone a standard PPV. C_3_F_8_ or 5500 cSt SO was used as endotamponade. As an intravitreal tamponade, SO (5500 cSt) was used in nine eyes and seven eyes used gas C_3_F_8_ tamponade. | To compare the surgical outcome between the group using SO and gas as tamponades. | Only one patient (5.3%) present with GRT | Not stated | 19 | 20 |
| Baba *et al*., 2021 [63] | Japan | A prospective, nationwide,  multicenter, cohort study | 26 institutions located throughout Japan | Data were obtained from the Japanese Retina and Vitreous Society ranged from 2016 to 2017 were used. Japanese individuals  >40 years of age were included, Eyes with PVR, and the primary outcome was the percentage of eyes that achieved 20/25 vision. | Patients younger than 40 years old, undergone previous surgeries for RRD, and cases with MHs, posterior  breaks and vitreous hemorrhage. | PPV was the most common procedure (1738, 79%), followed by SB (344, 16%) and PPV+SB (110, 5%). During the time of this registry, 99.5% of PPV procedures used MIVS with the use of 23-g, 25-g and 27-g systems. | Not applicable | Yes, but the exact number of cases were no stated. | Six months | Not stated | 2192 |
| Hocaoglu *et al*., 2019 [64] | Turkey | A retrospective case series | Istanbul Retina Institute | Patients of all ages who underwent primary 23-g PPV with injection of SO for GRT-related RRD from between December 2006 and June 2016 were included. | Patients with previous vitreoretinal surgery, penetrating ocular trauma, inadequate follow-up and incomplete data were excluded. | All patients underwent standard PPV. The mobile retina was flattened and stabilized by instillation of PFCL or by fluid-air exchange followed by internal drainage of subretinal fluid through either the edges of the GRT or a drainage retinotomy. Finally, surgery was completed by either air–oil or PFCL–oil (to obviate slippage of the posterior edge of the tear) exchange with 1000 cSt SO | Not applicable | 16 (36%) had a GRT ≥180° at baseline. 16 eyes (36%), have 90° tears, 11 eyes (28%) have tears >90° and <180°, and 16 eyes (36%) have tears ≥180°. | Mean = 37 ± 35 months | 42 | 45 |
| Minihan *et al*., 2001 [65] | The United Kingdom | A single center, retrospective cohort study | St Thomas’s Hospital | All patients who had surgical  repair of primary RRD between 1979-1980 to 1999. | Not stated | In the year 1979-80, 1 case (1%) was treated with PPV, while in the year 1999, 79 cases (63%) were treated with PPV. For the surgical procedure involving PPV with SO, 32 cases (25%) were conducted in 1999, whereas no cases were done in 1979-80, 23 of whom had preoperative PVR. | To compare the surgical approaches and success rate of two cohorts from 1979-80 and 1999. | Nine out of 23 cases (39.1%) of PPV with SO tamponade have GRT-related RRD. | Not stated | 124 patients in 1979–80 and 126 patients in 1999 | 124 cases in 1979–80 and  126 in 1999 |
| MacCumber *et al*., 2002 [66] | The United States of America | A retrospective noncomparative case series. | Not stated | 15 consecutive eyes of 15 patients with PVR, RD with GRT, or RRD between 1997 to 1999 were included. | Not stated | All 15 patients underwent PPV and PPL with preservation  and polishing the anterior capsule. A 20-g microvitreoretinal blade was used. Seven of 15 eyes (46.7%) had gas tamponade, and eight of 15 (53.3%) had SO tamponade placed at the time of vitrectomy. Three eyes that had gas tamponade had subsequent PCIOL placement. Five eyes with SO tamponade had subsequent silicone oil removal and PCIOL placement | Not stated | Yes, but the exact number of cases were no stated | > six months in all patients and averaged 11.5 months. | 15 | 15 |
| Goezinne *et al*., 2008 [67] | Netherlands | A retrospective case series | Department of Ophthalmology, University Hospital Maastricht, The Netherlands | All cases with GRT which were operated with PPV between March 1998 and August 2003. | Not stated | A standard PPV with a trocar microcannula system using a noncontact wide angle panoramic viewing system was performed in all the cases. All cases were operated with the use of PFCL and exchanged for SO (1000 cSt) or C_3_F_8_ gas at the end. During the operation an encircling SB was placed in 21 eyes (70%), 29 eyes (96.6%) received a SO tamponade, and one eye had a tamponade with C_3_F_8_ gas (3.4%). | Not applicable | All the 30 presented with GRT. The mean size of the GRT was 3.75 clock hours (range three – six clock hours). | Mean = 49 months (range 13–101 months) | 30 | 30 |
| Chehade *et al*., 2021 [68] | Australia | A retrospective case series | Either the Royal Adelaide Hospital  in Australia or the Adelaide Day Surgery | RRD patients with 18 years old and above, underwent PPV between November 2012 and August 2018 were included. A  100% fill PFD was used as a medium term post-operative tamponade. | Incomplete case notes were excluded from this study | Intraoperatively, a conventional PPV 25-g (32 cases, 37.6%) or 27-g (53 cases, 62.4%) vitrectomy was performed with noncontact wide-field  viewing system, with initial retinal reattachment, followed by the leaving PFD as a medium-term PFCL tamponade in all cases | Not applicable | 10 eyes (10.8%) | Mean = 16.0 ± 2.67 months (Range = three –59) | 85 | 85 |
| Bhurayanontachai *et al*., 2021 [69] | Thailand | A retrospective cohort study | Faculty of Medicine, Prince of Songkla University,Songkhl, Thailand | In a tertiary referral university hospital, patients with RRD from any cause, TRD, and any RRD or TRD that had previously failed treatment, underwent PPV with postoperative PFCL tamponade (with or without SB procedure). | The study excluded patients with other eye conditions like advanced glaucoma or uveitis, dense cataracts, systemic diseases, pregnancy, or missing critical data. Additionally, patients who needed another surgery to repair the detached retina with the use of PFCL were also excluded from the study. | The patients received 20- or 23-g PPV with PFCL tamponade. As much PVR as possible was removed before flattening the retina by PFCL. After one to two weeks, either C_3_F_8_ gas or SO tamponade was chosen for replacing  PFCL in the second operation. When PFCL was removed, 101 eyes (82.8%) were replaced by SO and 21 eyes (17.2%) were replaced by gas. | Not applicable | 27 eyes (22.1%) | Mean = 101.6 (±101.0) weeks | 121 | 122 |

AL, axial length; BCVA, best-corrected visual acuity; cTs, centiStokes; C_2_F_6_, hexafluoroethane; C3F8, perfluoropropane; C_10_F_18_, perfluorodecalin EVRS; DF, double filling; European vitreoretinal society; ERM; epiretinal membrane; g, gauge; GRT, giant retinal tear; IOL; intraocular lens; MHRD, macular hole retinal detachment; MH, macular hole; MIVS, microincision vitrectomy surgery; PPL, pars plana lensectomy; PFD, perfluorodecalin; PCIOL, posterior chamber intraocular lens; PDR, proliferative diabetic retinopathy; PFCL, perfluorocarbon liquid; PR, pneumatic retinopexy; PPV, pars plana vitrectomy; PVR, proliferative vitreoretinopathy; PFCL, perfluorocarbon liquid; PR, pneumatic retinopexy; PDMS, polydimethylsiloxane; RD, retinal detachment; RRD, rhegmatogenous retinal detachment; SF_6_, sulfur hexafluoride; SO, silicone oil; SB, scleral buckling; SD, standard deviation; TRD, tractional retinal detachment; VA visual acuity; VWTPL, vitrectomy without the intraoperative use of perfluorocarbon liquids

**Table S3.** Ages, Pre-operative PVR, Important Definitions, Anatomic Success, Functional Success, Complications, Risk Factors, and Conclusion

| **References** | **Mean Age ± SD, Years (Range)** | **Preoperative PVR** | **Important definitions** | **Anatomic success** | **Functional success** | **Complications** | **Risk factors** | **Conclusion** |
| --- | --- | --- | --- | --- | --- | --- | --- | --- |
| Sung *et al*., 2020 [36] | Mean = 51.73 years | 16 (36.4%) eyes in the total RRD group, and 6 (6.8%) eyes in the partial RRD group.  16.7% of the total 132 eyes were presented with PVR. | This study classified the detachment of the retina across all four quadrants as total RRD. The control group was defined as having less detachment, which was termed partial RRD. The PAS of the treatment was considered as the reattachment of the retina after one operation, which persisted for at least six months. Treatment failure referred to the retinal detachment recurred in any area. | The PAS rate was 96.6% in the partial RRD group, which was significantly higher than that of total RRD group (75%) In the total RRD group which achieved retinal reattachment, 84.8% underwent PPV only, while remaining 15.2% had underwent combined PPV and SB. In the total RRD group, 11 out of 44 eyes failed to achieve attachment, and nine underwent a second surgery, retinal reattachment after second surgery was achieved in six eyes, the FAS rate was 88.63%. In the partial RRD group, three out of 88 eyes had recurrent detachment, and all underwent a second surgery. All patients achieved reattachment after reoperation. | For the total RRD, the BCVA in logMAR before surgery and final BCVA after surgery were 2.23 ± 0.45 and 1.88 ± 0.96. A better result was shown in the partial RRD group, in which the BCVA in logMAR before surgery and final BCVA after surgery were 0.82 ± 0.83 and 0.35 ± 0.52. | In the total RRD group, among the 11 eyes which failed to achieve attachment, new retinal breaks were found in four eyes (3.0%), PVR in one (0.8%), new MHs in two (1.5%), and reopening of prior MHs in two eyes (1.5%). | Old age, pseudophakic eye, and macular hole as the type of retinal break were highly associated with low success rate. | Additional surgical procedures should be considered to combine with vitrectomy to achieve better surgical outcome in total RRD patients. |
| Li *et al*., 2021 [10] | Median = 46 (Range = four-72) years | Grade C PVR were found in seven (14.6%) eyes | Anatomic success was measured by SSAS or occurrence of a single operation retinal reattachment, and FAS or occurrence of final retinal reattachment regardless of the number of surgeries. Functional success was represented by a change in VA from baseline to the most recent follow-up. | SSAS rate was 75% at three months and 65% at two years. FAS was achieved at final follow-up in all 48 eyes (100%). | Median visual acuity improved from 20/250 preoperatively to 20/60 at final follow-up, with 20 (44%) eyes of eyes achieving postoperative VA of 20/40 or better. | Among the 16 eyes (33.3%) with recurrent detachments, 10 eyes (20.8% of the total 48 eyes) were presented with PVR. | Risk factors for the development of GRT: history of trauma, high myopia, Marfan syndrome, inherited vitreoretinopathies, Stickler syndrome, prior RD in other eyes. | Contemporary repair of some of the most complicated GRTs,  including those associated with trauma, PVR, and children,  leads to high rates of anatomic and functional success |
| Oderinlo *et al*., 2020 [37] | Mean ± SD = 46.1  ± 14.1 years | Not stated | Occurrence of single operation retinal reattachment maintained for a minimum of three months as well as  at the last clinic visit and anatomic reattachment at last surgery maintained until last clinic visit. A good visual outcome was defined as a best corrected VA of 6/60 and better at least two months after surgery maintained until last clinic visit. | PAS was achieved with 73 eyes (92.4%) in the GRT group and 157 eyes (86.7%) in the group of other holes and tears. FAS was achieved in 75 eyes (94.9%) in GRT group, and 164 eyes (93.2%)  in the group of other holes and tears. | VA in 66 eyes (79.6%) in the GRT group was worse than 3/60, compared to 117 eyes (60.3%) in group of other types of retinal tears. Good visual outcome was achieved in 48 eyes (59.3%) | Not stated | Not stated | The use of small gauge vitrectomy and PFCL can improve the anatomic and visual outcomes of surgeries involved in GRT-related RRDs. |
| Ting *et al*., 2020 [38] | Mean ± SD = 42.5 ± 13.7 years | 17 (13.4%) eyes | At each post-operative time-point, functional success was defined as a BCVA of <logMAR 1.0, PAS was defined as a 360° attached retina without any endotamponade with only a single retinal reattachment procedure (excluding removal of SO) and FAS was defined as having a 360° attached retina without any endotamponade, regardless of the number of retinal reattachment procedures required. Failure referred to a persistently detached retina | For anatomical outcomes at postoperative year one, 89 (74.8%) eyes had PAS, while 119 (93.7%) eyes  had FAS. At postoperative year five,  87.5% with FAS rate | Preoperatively, out of 127 eyes, about half (48.8%) presented with VA > logMAR 1.0, while 28 (22%) had a presenting VA of logMAR <0.3. Postoperatively, the mean logMAR was 0.86 (0.89) at one year, and the median logMAR was 0.51. 88 (69.3%) eyes achieving <logMAR 1.0 and 49 (38.6%) achieving <logMAR 0.3. | Cataract progression (n = 36, 28.3%), ERM (n = 27, 21.3%), transient raised IOP (n = 22, 17.3%), and 11 (8.7%) developed PVR. | Worse presenting VA, 150 degrees of more of GRT, detached macula, and presence of PVR | The types of surgery (PPV vs combined SB/PPV) and the number of breaks did not affect the anatomic and functional outcomes. |
| Mikhail *et al*., 2017 [39] | Mean = 56.6 (Range = 9-90) years | Not stated | 1) PAS defined as retinal reattachment with single operation and no residual intravitreal tamponade after two months, 2) FAS defined as retinal reattachment with more than one operation and no residual SO tamponade, 3) failure defined as persistent RD anywhere or retinal reattachment with longterm SO tamponade and 4) final VA after a minimum of six weeks of follow-up. | The overall PAS rate of retinal reattachment was 86%. There was no significant difference in the PAS rate between the three different surgical procedures with success rate of 86%, 85% and 80% for PPV, SB and PR. Patients presenting with GRTs (3) had 100% success rate with a planned two stage procedure using PFCL as a short-term postoperative tamponade, with PFCL removal and replacement by gas or SO after seven days. The overall FAS rate of retinal reattachment was 95.8% without SO; an additional 3.7% (eight eyes) were reattached with SO. Less than 1% (two eyes) remained detached, one eye deemed inoperable and one eye for which reoperation was refused by the patient. | Overall mean visual acuity improved from 1.1 to 0.4 LogMAR postoperatively. In 27 eyes undergoing a SB, the VA improved from a mean of 0.7 at presentation to 0.4 at final review. For the 175 eyes with primary PPV, the mean presenting VA 1.2 improved to 0.5. | The complication for SB group was localized subretinal hemorrhage (one patient). Of the vitrectomy subset, the complications were cataract development in 39 (18.3%) eyes, temporary raised IOP in 10 (4.7%) eyes, persistent raised IOP with optic nerve damage, ERM formation was found in four (1.98%) patients. | PVR negatively affected the primary success rate | The current trend in RRD surgeries for increasing usage of PPV. |
| Ghasemi *et al*., 2017 [40] | Mean ± SD = 33.96 ± 20.19 (Range = four –76) years | Grade C PVR was present in 14 (22.6%) eyes. | Not stated | PAS after one PPV procedure was achieved in 45 eyes (72.58%). FAS was achieved in 61 eyes (98.4%) at last follow up. 14 eyes needed one, two eyes needed two and one eye needed three additional PPV surgeries. | Mean preoperative BCVA was 2.47 ± 0.79 LogMAR at baseline, and 1.64 ± 0.85 and 1.46 ± 0.75 LogMAR at one and three months after surgery, respectively. At the last visit, mean BCVA was 1.43 ± 0.92 LogMAR. BCVA improved one line or more in 70% and three lines or more in 68% of the patients, respectively. | 17 eyes (27.4%) needed repeated PPV due to recurrent RRD associated with PVR. | Rate of repeated PPV was significantly correlated with baseline PVR and placement of encircling episcleral band. | High surgical success can be achieved in patients with RD associated with GRT with single or multiple surgeries. |
| Lumi *et al*., 2016 [41] | Mean ± SD of different groups was, Group one: 58.5 ± 13.5 Group two: 62.7 ± 10.2 Group three: 51.6 ± 17.8 Group 4: 70.7 ± 9.5 | 91 (77.8%) eyes have PVR < grade C1, while 26 (22.2%) eyes have PVR ≥ grade C1. | Surgery was considered successful only in cases when retina remained attached at one-year follow-up after a single procedure in eyes treated with PPV and gas tamponade or one year after SO removal in eyes with PPV and SO tamponade | The overall retinal reattachment rate with single surgery was 94.0%. FAS rate was 97.4%. In the case of established PVR ≥ C1, the reattachment rate was not statistically different (92.6%) from eyes with no PVR (91.1%) irrespective of lens status. A statistically significant  difference was found between redetachment rates only between phakic eyes with gas tamponade compared to SO. Reattachment rate proved to be similar in both AL groups (≤24 mm and > 24 mm). | Not stated | Not stated | Age, gender, lens status (phakic or pseudophakic), preoperative PVR grade, and AL of the eye. | High PAS rate of primary vitrectomy for complex RRD with either gas or SO tamponade was achieved in phakic as well as pseudophakic eyes irrespective of AL of the eye |
| Barth *et al*., 2023 [42] | Mean ± SD = 52 ±10.2 (Range: 33-73) years | Not stated | Not stated | Not stated | Before surgery, the mean logMAR BCVA had been 0.2, equivalent to a Snellen's VA of 0.63. The preoperative BCVA did not statistically differ between the two groups (p = 0.605). In 11 of the 22 cases (50%), a severe loss of BCVA was noted postoperatively for no apparent reason. The patients in the unusual vision loss group noted the deterioration of BCVA on average 12 weeks after surgery. In nine cases, the visual decline occurred while the SO was in-situ. Two patients noted visual decline about 2 weeks after SO removal when the transient air tamponade had dissolved. In the comparison group, two eyes had mild loss of BCVA (one to two lines) due to subtle intraretinal edema or secondary posterior capsule opacification, three eyes kept a stable BCVA, and the other six eyes showed improved BCVA at follow-up. After SO removal, mean logMAR BVCA in the unusual visual loss group was 1.0, equivalent to a Snellen's VA of 0.1, and 0.2 in the comparison group, equivalent to a Snellen's VA of 0.63. | Not stated | Not stated | In this case series, the unexplained visual loss was 50% using SO tamponade. Vitreoretinal surgeons should thoroughly question the need for SO tamponade, inform their patients of possible unexplained visual loss and remove SO as early as possible |
| Murtagh *et al*., 2020 [43] | Mean ± SD = 54.66 ± 15.29 (Range = 16-89) years | 82 patients (91.11%) had grade C  PVR, two patients had grade B (2.22%) and six patients had  grade A or early PVR (6.67%).  14.7% of the total 613 eyes were presented with PVR. | PAS was defined as  the retina being documented as flat at six months post procedure  (Under SO or otherwise) by fundus examination by using a slit lamp and a Volk lens and/or binocular indirect ophthalmoscopy and no further retinal surgical intervention for detachment  during that 6-month period. Visual success was defined as a gain of greater  than 0.3 logMAR units (the equivalent of three Snellen acuity  lines) or a logMAR of 0.3 or better at six months post procedure. | A mean PAS rate of 88.58% (543 out of 613  eyes) was recorded across all procedures with 70 eyes  redetaching in the six month postoperative period. Of these,  54 (77.14% of total redetachments and 16.31% of total PPV  cohort) had a primary PPV, nine (12.86% of redetachments and  6.98% of total SB cohort) were SB and seven (10% of redetachments and 4.67% of total PPV-SB cohort) were  PPV-SB. The FAS rate with SO removed in  this cohort is 90.6%. | Presenting VA was recorded for 601 of the 613  eyes (failure to record by a junior doctor at presentation), with  a median logMAR BCVA of 1 and a mean of 1.16. At six months post RD surgery, the mean  logMAR VA was 0.63 and the median logMAR VA was 0.5. In total, 71.71% of eyes achieved visual success. This consisted of 189 eyes that had a final VA logMAR of 0.3 or better and 242 that gained 0.3 logMAR or greater. Of  the 23-g sclerostomy group, 86.53% (n = 212 out of 245 eyes) achieved anatomical success while in the 20-g group, 88.28% achieved anatomical success (n = 211 out of 239 eyes). | A perioperative complication was recorded in 18 (2.94%) of the patients: raised postoperative IOP (eight cases, 1.3%), iatrogenic  tears (four cases, 0.7%), limited suprachoroidal hemorrhage (two cases, 0.3%), aborted SB (two cases, 0.3%), and exposed suture causing irritation which  required intervention (two cases, 0.3%) | Parameters recorded were the laterality of surgery, gender of the patient, duration of symptoms, age of patient, macular attachment status at presentation,  VA at presentation, location of the retinal breaks or tears, number of clock hours of retina involved in the detachment, type of procedure undertaken, type of anesthesia administered (local or general), length of stay in hospital, perioperative complications, previous cataract surgery, history of trauma, high myopia (AL recorded as greater than 24 mm) and the surgical and visual outcome of the patients at six months post procedure. | Surgical techniques utilized are also  in line with international reports. |
| Al-Wadani *et al*., 2014 [44] | Mean ± SD = 35.8 ± 8.7 (Range =  Four –80) years | 56 (30.4%) eyes | FAS was defined as complete retinal reattachment at the end of the follow-up period. | The retina remained attached after SO removal in 178 of the 184 eyes (96.73 %) included in the study. The retina detached after SO removal in six eyes that were operated on  for RRD. Recurrence of RRD was observed at one, two, four, five, 13, and 28 months after SO removal. The FAS rate in eyes that underwent relaxing retinotomy was 88.3 % (38 out of 43 eyes). | The data for the VA of 10 patients with RRD was missing. Among eyes with RRD that had data for final visual acuity, 84 out of 140 eyes (60 %) achieved final VA of 20/200. | The cause of the  redetachment in the first five eyes was the development of severe posterior PVR. | Not stated | This study explains the advantages of SO removal. |
| Zanzottera *et al*., 2022 [45] | Average = 56 (Range = 18–85) years | 15 (100%) eyes | Not stated | At the final examination (at one to 22 months), eleven patients (73%) had a retinal reattachment and four patients (27%) presented with early (within one month) recurrence of severe, inferior, or diffuse PVR and RD after PFCL/SO exchange. | Preoperatively, the BCVA was CF, HM, or light LP in 13 eyes (86%), 20/400 in one eye, and 20/50 in one eye. At the final follow up visit (at four to 22 months), VA improved in six eyes (50%), remained stable in three eyes (25%) and decreased in three eyes (25%). BCVA was CF/HM/LP in four eyes (33%), 20/200 or less in four eyes (33%), and 20/50 or more in three eyes (25%). One eye turned blind | Macula pucker (27%), severe hypotony (one case), PFCL emulsification (one case) | Not stated | In the management of complex RD, small-g PPV, and DF endotamponade using WAVS was a well-tolerated and effective technique to preserve VA and achieve anatomical success. |
| Bai *et al*., 2022 [46] | Mean ± SD = 49.82 ± 14.31 (Range = 14 – 82) years | 285 eyes (89.1%) with PVR CP a-c, 145 eyes (45.3%) with PVR CA a-b. | The two types of retinal reattachment rates are PAS, which measures reattachment after the initial surgery, and FAS, which measures reattachment at the end of follow-up. The patients in the study had a fixed follow-up one week after the operation, but after that point, some patients did not visit the surgeon regularly. As a result, the data collected one week postsurgery was the most complete. | Amongst the 320 eyes subjected to VWTPL, 305 (95.31%) had retinal reattachment immediately after the operation. Among the 15 eyes whose retina was not reattached immediately after VWTPL, five received a second VWTPL, which reattached the retina, whereas the other 10 eye cases refused a second VWTPL and remained unchanged until the last follow-up. All 22 eyes with retinal redetachment underwent a second VWTPL, which reattached the retina in 19 eyes, whereas three eyes were remained detached in situ. A total of 13 eyes had failed retinal reattachment. Except for the 27 lost eye cases, the FAS rate was 95.56% (280/293). | The 320 eyes showed better visual acuity than those before surgery, after one week of post-operative follow up (1.96 ± 1.07 vs. 1.43 ± 0.92, logMAR). | Not stated | Postoperative VA showed that age, uveitis, recurrent RD, the number of detached retinal quadrant, PVR and preoperative logMAR VA correlated with VA one week after the operation. | VWTPL is an effective and efficient surgical treatment for complicated retinal detachments |
| Quiroz-Reyes *et al*., 2022 [47] | Mean ± SD = 43.0 ±13.0 (Range = 19–76) years | 17 (22.4%) eyes | Anatomic success was measured by SSAS, FAS or occurrence of final retinal reattachment regardless of the number of surgeries. Functional success was represented by a change in VA from baseline to the most recent follow-up. | Seventy-six eyes from 66 patients were included in the study. The clinical charts of consecutive patients diagnosed with GRT-related RRD who were surgically managed between January 2010 and January 2021 were analyzed. Only eyes with the retina attached and functional vision on the patients' last postoperative evaluations, regardless of the number of surgical procedures needed, were included. All of them (100%) were treated with the use of PFCL. Five patients (6.6%) had bilateral GRT-related RRD, 61 patients (80.3%) had a monocular condition, and 21 eyes (27.6%) had final BCVA of ≥ 20/40. | The postoperative visual acuity (0.56 ± 0.26 logMAR)  was significantly better than the preoperative visual  acuity (1.07 ± 0.61 logMAR). The mean final postoperative BCVA for tear magnitude was significantly larger than for tear >270º (p =.048). For the main effect of tear magnitude, the mean of postoperative BCVA for tear 180-270º was significantly larger than for tear>270º | PVR resulted in 31.57% of the eyes | The management of GRTs is significantly challenging due to the presence of high risk intra- and postoperative complications and the high rate of recurrent RRDs due to the appearance of PVR that reaches an incidence between 40% and 50% with acute alterations in IOP due to uveal dysfunctions and a pro-cytokine inflammatory cascade from the blood-retinal barrier. | GRTs-related RRD has a guarded functional prognosis |
| Oderinlo *et al*., 2012 [48] | Mean ± SD = 46.1 ± 14.5 (Range = 10 - 69) years | PVR of grade C-1 in 30 (29.1%) eyes. | PAS was defined as retina reattachment  following the first surgery and maintained for at  least three months without additional surgical interventions.  FAS was defined as attached retina at least three months after the latest intervention, regardless of the  number of interventions. Visual outcome was defined as the best corrected VA at least 2 months after the last operation. A visual outcome of 6/60 and better was  considered good. | PAS was achieved in 83 eyes (80.5%), while FAS was achieved in 93 eyes (90.2%). | Compared to 88 eyes (87.1%) that had VA worse than 6/60 preoperatively only 59 eyes (58.4%) had  visual outcome worse than 6/60. A good visual outcome was  achieved in 40 eyes (38.9%). When compared to preoperative  VA, visual outcome improved in 61 eyes (59.2 %),  remained the same in 28 eyes (27.5%) and was worse in 14 eyes (13.7%). | Not stated | Absence of PVR grade C-1 and worse (p=0.015),  preoperative VA of 6/60 and better (p=0.037) and achievement of PAS (p=0.052)  significantly affected visual outcome positively. | Good visual outcome after surgery for RRD is influenced by preoperative VA of 6/60 and better, absence of PVR of grade C-1 and worse, as well as the achievement of PAS. |
| Ambiya *et al*., 2018 [49] | Mean ± SD = 46.89 ±18.04 years | 6 (4.51%) eyes | FAS was defined as total retinal reattachment after one or multiple surgeries, and anatomical failure as total or partial detachment of retina. | Out of the 133 cases of recurrent RD, 15 cases were not reoperated because of extremely poor visual prognosis. Of the remaining 118 cases, 92 underwent one  reoperation with anatomical success in 76 (82.61% of 92); 24 underwent two reoperations with FAS in 11 (45.83% of 24); and two underwent three reoperations with FAS in none. | There was a statistically significant improvement in  the BCVA of the 118 cases of recurrent RD that underwent one or multiple reoperations, from a mean value of  1.79 ± 0.46 logMAR (Snellen: 20/1233) to 1.32 (±0.60)  logMAR (Snellen: 20/418), P < 0.0001. However, only 5.9% (7/118) cases had final BCVA of ≥20/60; 28.81% (34/118) cases had <20/60–20/200, and the remaining  65.25% (77/118) cases had final BCVA <20/200. | Not stated | At the time of primary RD, eyes with PVR and PVR ≥ Grade C were less likely to have final BCVA ≥ 20/200. | PVR ≥ Grade C and multiple reoperations are associated with higher incidence of anatomical failure in recurrent RD surgery |
| Tabandeh *et al*., 2019 [50] | Mean = 60.8 years (Range = 19-91 years old) | Yes, the exact number of cases was not stated. PVR ranged from none to closed-funnel total RD. | Not stated | The retina was reattached with one procedure in 296 (95%)  eyes. SSAS rate was 95.2% for the eyes  with SB and 94.7% for the eyes without SB (p=1.0). FAS was achieved in 310 (99%) eyes. The SSAS rate was 92.8% for the eyes with GRT and 94.9% for eyes without  a GRT (p=0.53). FAS was achieved in 310 (99%) eyes. | The BCVA at the baseline was >20/40 in 76 (24%) eyes, 20/50–20/100 in 48 (15%) eyes, 20/200–20/400 in 46 (15%) eyes and <20/400 in 142 (46%) eyes. The mean logMAR equivalent was 1.40. At the last follow-up, the BCVA was >20/40 in 168 (54%) eyes, 20/50–20/100 in 60 (19%) eyes, 20/200–20/400 in 49 (16%) eyes and <20/400 in 35 (11%) eyes. Two eyes had no LP. The mean logMAR equivalent was 0.61 for the entire group. | Not stated | Not stated | Small gauge PPV without scleral-depressed shaving of the vitreous base  may be associated with good visual and anatomical outcomes. |
| Ali TR. 2014 [51] | Mean ± SD = 38.88 ± 21.45 (Group one) and  38.09 ±16.36 (Group two) | Yes, but the exact number of cases was not stated. | Not stated | Among the 43 group one patients who had the PFO being exchanged with SO in the same surgical procedure, six patients (14%) have detached retina. On the other hand, seven out of 22 group two patients (3%), who had the PFO being exchanged after three days of primary surgery, had detached retina after six months of follow-up. | Mean preoperative BCVA in group one was 2.02 ± 0.58 logMAR and in group 2 was 2.01 ± 0.53 logMAR. The logMAR VA in group one improved to 1.76 ± 0.43 logMAR after 1 month (1/60 on Snellen) and to 1.62 ± 0.62 logMAR after 6 months (3/60 on Snellen). This VA in group two improved to 1.85 ± 0.42 logMAR after one month (1/60 on Snellen) and 1.90 ± 0.72 logMAR after six months (<1/60 on Snellen). | No significant  complication  found after retaining  PFO for three days. | Not stated | PFO is efficacious and safe as a short-term vitreous substitute in primary RRD repair cases with inferior/multiple  breaks or GRTs or with extensive PVR. But keeping PFO for three days does not  significantly reduce the risk of redetachment with complex RRD cases. |
| Haugstad *et al*., 2017 [52] | 5.8% of participants aged 0–40  , 10.7% aged 41–51,  63.6% aged 51–70, while 19.9% aged  >70. | Not stated | PAS was defined as retinal reattachment six months after primary surgery without reoperation. In cases with SO removal, it was defined as reattached retina six months after oil removal. Laser postoperatively was not registered as a reoperation. In cases with no SO removal, attached retina under SO six months after primary operation was registered as success. FAS was defined as retinal reattachment six months after primary surgery including reoperations. | The PR group was excluded from this analysis because of a low number of patients (n = 2). Anatomical success rates were high in all three groups with primary and final failure rates of 11.0% and 1.9% for PPV, 13.0% and 0.0% for PPV-SB and 14.3% and 0.6% for SB. | Not stated | Not stated | Primary failure rate in patients with a RRD exceeding six clock hours was 19.7% compared to 9.6% in patients with smaller detachments, a failure rate of 15.2% in the group with VA <0.5 compared to 7.9% in the group with VA ≥0.5, a higher failure rate for patients older than 70 years compared to patients 70 years or younger (18.4% versus 10.9%). | No significant differences in the PAS and FAS rates between PPV, PPV-SB and SB in the treatment of RRD. |
| Scheerlinck *et al*., 2018 [53] | The median age (range) for each of the groups were: 62 (45 to 72) for macula on, gas tamponade: 62 (30 to 74), macula on SO tamponade 62 (54 to 75), macula-off gas tamponade 61 (51 to 74) for macula off SO tamponade | PVR grade C1 was present in one eye with a macula-on GRT | Not stated | Not stated | For macula on RRD, the preoperative BCVA for gas tamponade and SO tamponade were logMAR 0.11 and 0.2, respectively. Macula off RRD, on the other hand, has preoperative BCVA for gas tamponade and SO tamponade, as logMAR 1.78 and 1.30, respectively. For macula on RRD, the post-operative BCVA for gas tamponade and SO tamponade were logMAR 0.08 and 0.16, respectively. Macula off RRD, on the other hand, has postoperative BCVA for gas tamponade and SO tamponade, as logMAR 0.26 and 0.39, respectively. | Not stated | Not stated | The mean postoperative BCVA tended to be worse after SO tamponade, both for macula on and macula off RRD, this was not statistically significant in both groups. |
| Shu *et al*., 2019 [54] | Mean ± SD = 33.0 ± 11.8 (Range = 2–49) years | Preoperative PVR cases were excluded | Not stated | The PAS rate for all eyes was 93.1%: 92.2% for the SB group and 93.9% for the PPV group. The FAS rate for all eyes was 99.0%: 99.3% for the SB group and 98.9% for the PPV group, with no statistically significant difference between the two groups | Improvements of BCVA were noted. The preoperative BCVA for all patients was 0.52 ± 0.73 logMAR. Final BCVA (the best postoperative BCVA) was 0.17 ± 0.45 logMAR. | PVR (1.3%), endophthalmitis (0.3%), macular pucker (1.7%), cataract formation (3.9%), elevation of IOP (0.01%) and eye movement disorder (0.002%) were observed. | Preoperative BCVA, forms  of tears, locations of tears, atrophic holes, and GRT. | This study suggested that SB is a better choice in some cases, even when MIVS is available. In young patients without PVD, SB can be recommended; in young patients with PVD, PPV can be recommended with the expectation of improved PARR and complications. |
| Eiger-Moscovich *et al*., 2017 [55] | Mean ± SD, 54 ± 12 (Range = 30 to 79) years | Not stated | Not stated | Retinal reattachment was achieved intraoperatively in all patients. Repeated detachment with PVR  occurred in one patient (8%), who underwent repeated vitrectomies. At the last follow-up visit, the retina was attached in all patients. | The macula was detached at presentation in three patients, all  of which showed an improvement in BCVA postoperatively  compared with preoperatively, and the final BCVA was equal  to or better than 20/100. Of the 10 patients in whom the macula was attached at presentation, six (60%) had a final  BCVA that was equal to or better than the initial BCVA. | Postoperative elevation in IOP was measured in two patients (15%), PFCL was noted in the anterior chamber of one patient, cataract developed in two patients (15%), and CME in another two (15%). | Not stated | PFCL is a safe and effective material for short-term vitreoretinal tamponade following vitrectomy for giant retinal tear |
| Adelman *et al*., 2013 [70] | Not stated | Grade B PVR (n = 917, 11.9%); grade C-1 PVR (n = 637, 8.3%) | The failure rate was divided into three categories: level one represented the true failure rate of eyes with detached retina judged to be inoperable by the end of the study, level two represented the percentage of eyes with SO remaining in the eye at the end of the study, and level three represented the percentage of eyes that experienced a recurrence of RD or complications after the initial procedure, requiring additional surgery. | Treatment with vitrectomy resulted in a lower level one failure rate. Application of a supplemental SB when vitrectomy was performed was  associated with a significantly higher level one failure rate when compared with eyes where no SB was placed. When tamponade with either gas or SO was evaluated, the level one failure rate was not statistically different  between the two groups, whereas the level two failure rate was higher  in the SO group. | Not stated | Not stated | Choroidal detachment, significant hypotony, level of PVR (Grade A, B, and C-1), size of the largest retinal break, number of detached quadrants, lens status, total number of tears, and presence of posterior breaks including MHs. | This study demonstrated that  when choroidal detachment, significant hypotony, a large  tear, or a GRT is present, then vitrectomy is the procedure  of choice. |
| Li *et al*., 2020 [56] | Mean ± SD = 54.47 ± 11.81 years | PVR C1 in two (3.4%) eyes and B or  below in 57 (96.6%) eyes. | Not stated | The PAS and FAS rates were 94.9% (56/59) and 98.3% (58/59), respectively. Of the three eyes which  developed redetachment of the retina, one eye had postoperative progression of PVR, and two eyes were RRD associated with MH in high myopia. Reattachment was achieved after the second surgery in these eyes, and the retina remained attached and stable after SO removal. The other case of redetachment was related to postoperative progression to severe PVR one month after  surgery. Subsequent surgery was performed using SO tamponade, but the retina was only partially reattached after SO removal seven months later. At the end of the latest follow-up, 58 (98.3%) eyes achieved reattachment of the retina, and the remaining one eye still had localized detachment due to PVR recurrence  postoperatively. All the three eyes with GRT achieved PAS with air tamponade | Postoperative BCVA significantly improved from one week onwards after surgery (preoperative: 1.28 ± 0.79 logMAR 20/381 Snellen equivalent; 1-day: 2.12 ± 0.21  logMAR 20/2,636 Snellen equivalent; 1-week: 1.00 ±  0.57 logMAR 20/200 Snellen equivalent; one month: 0.66  ± 0.47 logMAR 20/91.4 Snellen equivalent; three months: 0.63 ± 0.47 logMAR 20/85.4 Snellen equivalent.  six-month: 0.54 ± 0.42 logMAR 20/69.4 Snellen equivalent; P: 0.001). | Serous choroidal detachment (five eyes, 8.5%), ERM formation (four eyes, 6.8%), secondary  cataract surgery was performed in 13 of the 53 phakic eyes (24.5%) during follow-up. | Not stated | Modern small-gauge PPV with air tamponade  could achieve clinically acceptable surgical outcomes  in the management of primary RRD of short duration,  predominantly superior breaks, with three or fewer quadrants involvement, and PVR grading B or below, possibly even for selected cases with GRT. |
| Abdala-Caballero *et al*., 2022 [57] | Mean = 49 years  (Range = 18–65) | PVR grade was B in 14 eyes (44%),  in second place C1 with 8 cases (26%), followed  by A (11%) and C3 (11%) with four eyes each  one, and C2 present in 2 cases (7%). | Not stated | A PAS rate of 93% was obtained with the performed technique.  In the cases with retinal re-detachment, a second surgery was needed, obtaining 100% FAS rate | Mean preoperative BCVA was 20/800 — ≥ 20/400  in 29 eyes (93%).  At six months, only one eye (4%) had BCVA ≥ 20/40, and 16 eyes (50%) had  BCVA ≥ 20/400. At one-year of follow-up, one  eye (4%) had BCVA ≥ 20/40, 23 eyes had between 20/40–20/200, and eight eyes (22%) had BCVA ≥ 20/400. Compared with BCVA at initial presentation, 29 eyes (93%) were stable or improved  at final follow-up. | Not stated | The three eyes (7%) that required two surgeries  presented retinal re-detachment, and two of them had a higher degree of presurgical PVR (grade C  compared to lower grades; p = 0.065). | Initial surgical management, as we presented in  the presence of RD secondary to GRT, is excellent election to achieve high rates of retinal reattachment  and reduces the need for a second surgery |
| Ghosh *et al*., 2004 [58] | Mean = 35.03 years (Range = seven -60 years) | Not stated | Not stated | PAS was achieved in 19 patients (65.51%). In all, 10 patients (34.48%) needed more than one surgery to reattach the retina. Of these, six were reattached with one further surgery. Four patients had total retinal detachment with severe PVR and needed between two and three additional surgical procedures. These four patients had persistent RD even after multiple surgeries. The retina was anatomically attached in 25 cases (86.20%) on their last postoperative visit. | Preoperative BCVA ranged from LP in patients with macular detachment to 6/9 in patients with localized RD not involving the macula. Postoperative BCVA improved in 22 patients (75.86%) with a range from 6/60 to 6/9 and deteriorated in five patients (17.24%) with persistent RD and extensive PVR. Two patients (6.9%) did not have any change in their visual status. There was an improvement of two or more Snellen's lines in nine patients (31.03%). | One lens trauma (3.4%) and one iatrogenic GRT (3.4%), Seven patients (24.1%) developed cataracts postoperatively, and four (13.8%) had extensive RD and PVR after multiple surgery and had a quiet eye. | Not stated | With newer techniques of surgical treatment of GRTs, the anatomical and visual outcomes are more favorable. |
| Kumar *et al*., 2018 [59] | Mean = 25.7 years | 76.5% (n = 13/17) of eyes have grade B PVR, one eye had PVR grade A. | The functional outcome of surgery was evaluated by BVCA at the last follow-up. PAS was primary retinal reattachment without any reoperation at the last follow-up. | PAS was  attained in all eyes. FAS rate was  achieved in 88.2% of eyes (n = 15/17). | Only 35.2% of eyes achieved final VA ≥20/80 | PVR (two eyes, 11.7%), inferior traction (one eye, 5.9%), Persistent corneal epithelial defect (one eye, 5.9%) corneal  decompensation (one eye, 5.9%), cataract (four eyes, 23.5%), and secondary glaucoma (two eyes, 11.7%). | Not stated | 25-g PPV can achieve excellent retinal reattachment rates in GRT-related RRD without intraoperative and  postoperative complications. |
| Schwartz *et al*., 2002 [60] | Not stated | No. | Not stated | 186 eyes (82%) achieved reattachment with one SB procedure, an additional 30 eyes (13%) achieved reattachment after one or more vitreoretinal procedures, while 11 eyes (5%) detached at 20 years of follow up. For the four eyes with GRT, no eyes achieved retinal reattachment with one SB procedure, one eye never achieve reattachment despite one additional vitreoretinal surgery, the other three eyes achieved reattachment after one additional surgery, with a median 20 years of visual acuity of 20/200 (range 20/25 to CF) | 186 eyes (82%) achieved final VA of 20/40, an additional 30 eyes (13%) achieved VA of 20/50 with one or more vitreoretinal procedures, while 11 eyes (5%) have final VA of no light perception at 20 years of follow up. | choroidal detachment (one eye, 0.8%), orbital cellulitis (one eye, 0.8%) | Not stated | Stability could be achieved in RRD patients using SB as their surgical options. |
| Filippelli *et al*., 2021 [61] | Mean = 65.8 ± 11.4 (Range =23–87) years | PVR was grade A in seven (15.9%), grade B in 28 (63.6%), and grade C in 9 (20.5%) eyes. | PVR was defined as follows: grade A: vitreous haze and pigment; grade B: wrinkling of the inner retinal surface, retinal stiffness, rolled edge of breaks, and vessel tortuosity; and grade C: full-thickness and fixed retinal folds. VA of CF was converted to 1.4, that of HM was converted to 2.7, and that of LP was converted to 3.7 logMAR. | In both eyes with retinal vessel printings, detachment involved four quadrants in one case (breaks located in the upper and lower quadrants) and three quadrants in the other (with the break located in the upper quadrants) | Preoperative BCVA was 2.1 ± 1.0 logMAR and improved to 0.8 ± 0.7 logMAR at the last follow-up. | A bleb of SO migrated in the anterior chamber in two eyes (4.5%), macular edema developed in three eyes (6.8%), and an IOP ≥ 22 mmHg was recorded in seven eyes (15.9%). One month after the first operation, retinal vessel printings located inferiorly to the retinal vessels (indicative of upward displacement) were noted in 2 eyes (4.5%). | Not stated | PPV with SO injection for complicated RRD repair is associated with good anatomical and functional outcomes. Furthermore, the rate of unintentional retinal displacement is very low. |
| Christensen *et al*., 2012 [62] | For group of using gas as tamponades, mean = 62 (range = 47–75) years. While for the group of using SO as tamponades, mean = 51 (rang = 33–69) years | Preoperative PVR grades A and B were seen in both groups, but the exact number of cases were not stated. | Not stated | Not stated | Both groups with gas tamponade and SO tamponade patients had identical mean preoperative VA of 79 ETDRS letters (6/7.5 Snellen). Median postoperative VA at follow-up was significantly poorer in SO eyes (58 letters, >6/24 Snellen) compared to gas eyes (83 letters, >6/7.5 Snellen. Three of nine (33%) SO eyes had a severe reduction in final BCVA to ≤36 letters (6/60 Snellen) and six of nine (67%) had final BCVA ≤71 letters (6/12 Snellen). No gas eyes had final BCVA below 77 letters (6/9 Snellen) | Not stated | Not stated | Severe visual loss after SO use was observed in 1/3 of patients |
| Baba *et al*., 2021 [63] | A minimum of 40 years old | Yes, but the exact number of cases were not stated | The lower VA of no LP were graded as 4.0 logMAR units, light perception 3.0 logMAR units, HM 2.3 logMAR units and CF 2.0 logMAR units. PAS was defined as having no additional surgery during the six postoperative months. | The overall FAS success was 90.8%; PAS in cases treated with SB was 93.1%, PPV was 91.8% and PPV+SB was 68.7%. | The surgical outcomes were improved, as mean total baseline BCVA improved from 0.52±0.78 to six months post-operative BCVA of 0.09±0.31 logMAR. Baseline BCVA were 0.15±0.48,  0.57±0.79, and 0.87±0.95 logMAR units after SB alone, PPV alone, and combined PPV and SB, respectively.  Postoperative BCVA were 0.03±0.22,  0.08±0.29, and 0.32±0.45 logMAR units after SB alone, PPV alone, and combined PPV and SB, respectively. The cases with a high level of failure had poorer postoperative BCVA  and lower possibility of achieving a BCVA of 0.1 logMAR units in cases treated by PPV alone. None of the cases treated by SB had level three failure cases but had the same trend. | Not stated | Older  age (>70 years), low IOP (<10mm Hg), high myopia (>−5 diopters), multiple retinal breaks (> four), GRT (>90°), wide RD (> three quadrants) and macula-off RD were  associated with less probability of postoperative 20/25 vision. | Better BCVA in patients with RRD who had undergone PPV was observed. However, if  concurrent cataract surgery is not performed, BCVA will be comparable with either PPV or SB. |
| Hocaoglu *et al*., 2019 [64] | Mean ± SD = 43.3 ± 13.1 (Range = 9–62) | 19 eyes (42%) were found to have some degree of PVR (range, Grade A–C). Among these, seven eyes (16%) have grade C PVR. | PAS was defined as a fully attached retina for a minimum of six months after SO removal, without the need for additional surgery or laser photocoagulation. | The PAS rate was 84%, and the overall FAS rate was 98%. | The preoperative mean ± SD visual acuity was 1.5 ± 1.3 logMAR, which is equivalent to Snellen visual acuity of 20/580. The mean postoperative VA was 0.46 ± 0.62 logMAR. The final VA ≥20/40 was achieved in 64%. | Transient elevation of IOP in 10 (22%) of eyes, cataract formation (five out of nine phakic eyes), ERM formation four (9%) eyes. | Not stated | The results of this study support management of GRT-related RRD using modern WAVS and improved instrumentation with vitrectomy alone in a lens-sparing approach. |
| Minihan *et al*., 2001 [65] | Not stated | In 1979–80 32 eyes, and in 1999 24 eyes had preoperative  PVR.  22.4% of the total 250 cases were presented with PVR. | Not stated | The FAS rate was 88.7% (110 of 124) and 93.6% (118 of 126), for 1979-80 and 1999 cohort, respectively | Median preoperative VA in 1979–80 and 1999 was counting fingers. Median postoperative VA was 6/24 in 1979–80 and in 1999. In 1979–80. 31% of cases had a final VA of 6/12 or better,  while 47% did in 1999. | Postoperative cataracts (18 cases in 1999 versus five in 1979-80) | Not stated | RRD that was treated by PPV had increased compared with 20 years ago. |
| MacCumber *et al*., 2002 [66] | Not stated | Grade C PVR was present preoperatively in 11 and was anterior in 5, the remaining eyes (n=4) have grade A PVR. | Not stated | 14 (93.3%) eyes have complete reattachment at the final visit. | The preoperative VA was CF in four (26.7%) eyes, HM in five (33.3%) eyes, LP in two (13.3%) eyes, 20/400 in two (13.3%) eyes, 20/250 in one (6.7%) eye, and 20/200 in one (6.7%) eye. The postoperative acuity was CF in three (20%) eyes, 20/400 and 20/70 in two (13.3%) eyes, and one (6.7%) eye each for HM, 20/250, 20/200, 20/80, 20/60, and 20/50. Final VA was better or equal to preoperative VA in all eyes, improving by four ± four lines overall. | Not stated | Not stated | Preserving the capsule helps prevent intraoperative and postoperative complications of gas or SO, simplifying future PCIOL placement, and maintains a normal iris appearance |
| Goezinne *et al*., 2008 [67] | Mean = 53.2 years (Range = 30–70  years) | 19 of the 30 eyes had preoperative PVR grade A (63.3%) and 11 PVR grade B (36.7%) | Anatomic success was defined as a  complete attachment of the retina. | PAS after one vitrectomy procedure in this series of 30 eyes was achieved in 21 eyes (70%), and ultimately FAS was attained in 29 (96.7%) eyes. | In this study, 43% of the eyes had a postoperative  VA of less than 0.1. In this study, 57% of the patients had a postoperative VA better than 0.1, and 60% had an equal or better postoperative VA than  preoperative, which is comparable to results reported  earlier by others. | The development of ERM of the macula with CME in three eyes (33.3%), postoperative endophthalmitis (one patient, 3.3%), high IOP (one patient, 3.3%), and corneal decompensation (one patient, 3.3%). | Encircling SB as risk factor for redetachment. | In conclusion, our study shows that a recurrent RD rate was observed in 30% of cases and that further surgical interventions finally resulted in an attached retina in 97% of the eyes. Vitrectomy with an encircling SB and SO tamponade seems to be a successful treatment for complicated RDs due to GRT. |
| Chehade *et al*., 2021 [68] | Mean ± SD = 61.7 ± 2.79 years | PVR-C in 26 (30.6%) eyes | PAS was defined as retinal reattachment with no residual tamponade at three month follow-up and no further surgery required  other than heavy liquid exchange. | FAS was achieved in 98.8% of cases. | The mean presenting BCVA was 1.57±0.20 logMAR, while the mean final BCVA was 0.94±0.20 logMAR. The mean improvement in BCVA of 0.64±0.20 logMAR, which was statistically significant. GRT was associated with a lower rate of improvement in BCVA, but a higher rate of  anatomical success, however, this was not statistically significant (p=0.488 and p=0.713). | CME in 14 patients (16.5%), PFCL in the anterior chamber in eight patients (9.4%), persistent elevated IOP  at three months or hypotony in three patients (3.5%) each and granulomatous inflammation in two patients (2.4%). In addition, 20 patients (23.5%) required cataract surgery within the follow-up period. | Not stated | Medium-term tamponade PFD appears to be an efficacious and safe technique for management of a variety of complex RDs, including those secondary to GRT, the presence of PVR-C, inferior location, tractional, traumatic, and re-detachments. |
| Bhurayanontachai *et al*., 2021 [69] | Mean ± SD = 42 ±18.5 years | Yes, but the exact number of cases were not stated | The primary outcome was reattachment of the retina (Anatomical outcome). The secondary outcomes were vision improvement (functional outcome). | 114 eyes (93.5%) had baseline vision worse than 20/200. The retinal reattachment rate was 80.3%. At one year, the retention probability of retinal reattachment  was 0.84. | The mean preoperative BCVA was 1.8 (±0.4) logMAR, while the mean postoperative BCVA was 1.6 (±0.7) logMAR. The visual improvement was found in 45.9% of patients, but the median of final vision was not different between baseline and the last visit. | Immediate postoperative IOP elevation which usually occurred the next day after PFCL placement was found in 51.2% of patients, IOP elevation and hypotony occurring after PFCL removal was found in 25.4 and 23% of eyes, respectively, optic nerve atrophy and cataract progression were equally found in 22.1% of eyes, ERM occurred in 13.1% of eyes, suspected late postoperative endophthalmitis in 0.8% patients. | Not stated | The rate of retinal reattachment operated with a short- to medium-term PFCL tamponade achieved a high satisfaction rate. |

AL, axial length; BCVA, best-corrected visual acuity; CF, counting fingers; CME, cystoid macular edema; DF, double filling; ETDRS, early treatment diabetic retinopathy study; ERM, epiretinal membrane; FAS, final anatomic success; GRT, giant retinal tear; HM, hand movement; IOL, intraocular pressure; logMAR, logarithm of the minimum angle of resolution; MH, macular hole, MIVS, microincision vitrectomy surgery; PARR, primary anatomic reattachment rate; PFD, perfluoro decalin; PFO, perfluoro-n-octane; PVR, proliferative vitreoretinopathy; PPV, pars plana vitrectomy; SB, scleral buckling; SSAS, single surgery anatomical success; SO, silicone oil; PAS, primary anatomic success; PCIOL, posterior chamber intraocular lens; PFCL, perfluorocarbon liquid; VA, visual acuity; SD, standard deviation; WAVS, wide-angle viewing systems; VWTPL, vitrectomy without the intraoperative use of perfluorocarbon liquid

**Table S4.** Quality Assessment of Case Series Using Joanna Briggs Institute Checklist for Case Series [26]

| **No.** | **Reference** | **Criteria** | | | | | | | | | | **Total score** |
| --- | --- | --- | --- | --- | --- | --- | --- | --- | --- | --- | --- | --- |
|  |  | **Clear inclusion criteria** | **Standard and reliable measurement of the condition** | **Valid methods for identification of the condition** | **Consecutive inclusion of participants** | **Complete inclusion of participants** | **Reporting of participant demographics** | **Reporting of clinical information** | **Reporting of outcomes or follow-up results** | **Reporting of presenting site/clinic demographic information** | **Appropriate statistical analysis** |  |
| 1. | Lee et al. [10] | 1 | 1 | 1 | 1 | 1 | 1 | 1 | 1 | 0 | 1 | 9 |
| 2. | Oderinlo et al [37] | 1 | 1 | 1 | 1 | 1 | 1 | 1 | 1 | 0 | 1 | 9 |
| 3. | Mikhail et al [39] | 1 | 1 | 1 | 1 | 1 | 1 | 1 | 1 | 0 | 1 | 9 |
| 4. | Ghasemi et al. [40] | 1 | 1 | 1 | 1 | 1 | 1 | 1 | 1 | 0 | 1 | 9 |
| 5. | Al-Wadani et al [44] | 1 | 1 | 1 | 1 | 1 | 1 | 1 | 1 | 0 | 1 | 9 |
| 6. | Zanzottera eta al[45] | 1 | 1 | 1 | 1 | 1 | 1 | 1 | 1 | 0 | 1 | 9 |
| 7. | Bai et al. [46] | 1 | 1 | 1 | 1 | 1 | 1 | 1 | 1 | 0 | 1 | 9 |
| 8. | Oderinlo et al. [48] | 1 | 1 | 1 | 1 | 1 | 1 | 1 | 1 | 0 | 1 | 9 |
| 9. | Tabandeh et al [50] | 1 | 1 | 1 | 1 | 1 | 1 | 1 | 1 | 0 | 1 | 9 |
| 10. | Elger-Moscovich et al [55] | 1 | 1 | 1 | 1 | 1 | 1 | 1 | 1 | 0 | 1 | 9 |
| 11. | Li et al.[56] | 1 | 1 | 1 | 1 | 1 | 1 | 1 | 1 | 0 | 1 | 9 |
| 12. | Abdala-Caballero et al. [57] | 0 | 1 | 1 | 1 | 1 | 1 | 1 | 1 | 0 | 1 | 9 |
| 13. | Ghosh et al.[58] | 1 | 1 | 1 | 1 | 1 | 1 | 1 | 1 | 0 | 1 | 9 |
| 14. | Kumar et al. [59] | 1 | 1 | 1 | 1 | 1 | 1 | 1 | 1 | 0 | 1 | 9 |
| 15. | Schwartz et al. [60] | 1 | 1 | 1 | 1 | 1 | 1 | 1 | 1 | 0 | 1 | 9 |
| 16. | Filippelli et al. [61] | 1 | 1 | 1 | 1 | 1 | 1 | 1 | 1 | 0 | 1 | 9 |
| 17. | Christensen et al. [62] | 1 | 1 | 1 | 1 | 1 | 1 | 1 | 1 | 0 | 1 | 9 |
| 18. | Hocaoglu et al. [64] | 1 | 1 | 1 | 1 | 1 | 1 | 1 | 1 | 0 | 1 | 9 |
| 19. | MacCumber et al. [66] | 1 | 1 | 1 | 1 | 1 | 1 | 0 | 1 | 0 | 1 | 8 |
| 20. | Goezinne et al. [67] | 1 | 1 | 1 | 1 | 1 | 1 | 1 | 1 | 0 | 1 | 9 |
| 21. | Chehade et al. [68] | 1 | 1 | 1 | 1 | 1 | 1 | 1 | 1 | 0 | 1 | 9 |

**Table S5.** Quality Assessment of Cohort Studies using Joanna Briggs Institute Checklist for Cohort Studies [25].

| **No.** | **Reference** | **Criteria** | | | | | | | | | | | **Total score** |
| --- | --- | --- | --- | --- | --- | --- | --- | --- | --- | --- | --- | --- | --- |
|  |  | **Similarity of groups** | **Consistency in measuring exposures** | **Validity and reliability of exposure measurement** | **Identification of confounding factors** | **Strategies to deal with confounding factors** | **Absence of outcome at baseline** | **Validity and reliability of outcome measurement** | **Reporting and sufficiency of follow-up time** | **Completeness and description of follow-up loss** | **Strategies to address incomplete follow-up** | **Appropriate statistical analysis** |  |
| 1. | Sung et al. [36] | 1 | 1 | 1 | 1 | 1 | 1 | 1 | 1 | 1 | 0 | 1 | 10 |
| 2. | Ting et al. [38] | 1 | 1 | 1 | 1 | 0 | 1 | 1 | 1 | 1 | 0 | 1 | 9 |
| 3. | Lumi et al. [41] | 1 | 1 | 1 | 1 | 1 | 1 | 1 | 1 | 1 | 1 | 1 | 11 |
| 4. | Barth et al. [42] | 1 | 1 | 1 | 1 | 1 | 1 | 1 | 1 | 1 | 0 | 1 | 10 |
| 5. | Murtagh et al. [43] | 1 | 1 | 1 | 1 | 1 | 1 | 1 | 1 | 1 | 0 | 1 | 10 |
| 6. | Quiroz-Reyes et al. [47] | 1 | 1 | 1 | 0 | 1 | 1 | 1 | 1 | 1 | 0 | 1 | 9 |
| 7. | Ambiya et al. [49] | 1 | 1 | 1 | 1 | 1 | 1 | 1 | 1 | 1 | 0 | 1 | 10 |
| 8. | Ali et al. [51] | 1 | 1 | 1 | 0 | 0 | 1 | 1 | 1 | 1 | 0 | 1 | 8 |
| 9. | Haugstad et al. [52] | 1 | 1 | 1 | 1 | 1 | 1 | 1 | 1 | 1 | 0 | 1 | 10 |
| 10. | Scheelinck et al. [53] | 1 | 1 | 1 | 1 | 1 | 1 | 1 | 0 | 1 | 0 | 1 | 9 |
| 11. | Shu et al. [54] | 1 | 1 | 1 | 1 | 1 | 1 | 1 | 1 | 1 | 0 | 1 | 10 |
| 12. | Adelman et al. [70] | 1 | 1 | 1 | 1 | 1 | 1 | 1 | 1 | 1 | 0 | 1 | 10 |
| 13. | Baba et al. [63] | 1 | 1 | 1 | 1 | 1 | 1 | 1 | 1 | 1 | 0 | 1 | 10 |
| 14. | Minihan et al. [65] | 1 | 1 | 1 | 1 | 0 | 1 | 1 | 0 | 1 | 0 | 1 | 9 |
| 15. | Bhurayanontachai et al. [69] | 1 | 1 | 1 | 1 | 1 | 1 | 1 | 1 | 1 | 0 | 1 | 10 |
